# Review of recent advances in post-harvest techniques for tropical cut flowers and future prospects: *Heliconia* as a case-study

**DOI:** 10.3389/fpls.2023.1221346

**Published:** 2023-07-28

**Authors:** Moumita Malakar, Patrícia Duarte de Oliveira Paiva, Margherita Beruto, Antonio Rodrigues da Cunha Neto

**Affiliations:** ^1^ Department of Horticulture & Floriculture, Central University of Tamil Nadu, Thiruvarur, India; ^2^ Departamento de Agricultura, Escola de Ciências Agrárias, Universidade Federal de Lavras, Lavras, MG, Brazil; ^3^ International Society for Horticultural Science (ISHS), Ornamental Plant Division, San Remo, Italy

**Keywords:** *Heliconia*, post-harvest, longevity, tropical ornamental cut flowers, preservation, packaging

## Abstract

Aesthetic attributes and easy-to-grow nature of tropical cut flowers (TCFs) have contributedto their potential for increased production. The dearth of information regarding agronomic practices and lack of planting materials are the key hindrances against their fast expansion. Unconventional high-temperature storage requirements and the anatomy of the peduncle contribute topoor vase life performance, while troublesome packaging and transport due to unusual size and structureprimarily cause post-harvest quality deterioration. Nonetheless, the exotic floral structuresconsequently increase market demand, particularly in temperate countries. This boosts studies aimed at overcoming post-harvest hindrances. While a few TCFs (*Anthurium, Strelitzia, Alpinia*, and a few orchids) are under the spotlight, many others remain behind the veil. *Heliconia*, an emerging specialty TCF (False Bird-of-Paradise, family Heliconiaceae), is one of them. The structural uniquenessand dazzling hues of *Heliconia* genotypes facilitate shifting its position from the back to the forefrontof the world floriculture trade. The unsatisfactory state-of-the-art of *Heliconia* research and the absence of any review exclusively on it are the key impetus for structuring this review. In addition to the aforementioned setbacks, impaired water uptake capacity after harvest, high chilling sensitivity, and the proneness of xylem ducts to microbial occlusion may be counted as a few additional factors that hinder its commercialization. This review demonstrates the state-of-the-art of post-harvest research while also conceptualizing the implementation of advanced biotechnological aid to alleviate the challenges, primarily focusing on *Heliconia* (the model crop here) along with some relevant literature on its other allied members. Standard harvesting indices, grading, and packaging are also part of the entire post-harvest operational chain, but since these phases are barely considered in *Heliconia* and the majority of tropical ornamentals except a few, a comprehensive account of these aspects has also been given. The hypothesized cues to nip chilling injury, resorting to different bio-chemical treatments, nano-based technology, and advanced packaging techniques, may help overcome preservation difficulties and propel its transition from niche to the commercial flower market. In a nutshell, readers will gain a comprehensive overview of how optimum post-harvest handling practices can rewardingly characterize this unique group of TCFs as the most remunerative component.

## 1 Introduction

The climate of tropical region (starts from Tropic of Cancer to the North to the Tropic of Capricorn to the South) gifts brightly hued tropical flowers (considered as niche products) which since time immemorial, receiving huge admiration (Yue and Hall, 2010) for their shape, symbolic as well as aesthetic significances. Traditionally, these plants are used in gardens and also as cut flower. The current scenario, post-pandemic, is associated with the increasing popularity of these segments among consumers in pan world (Paull, 1990; Paull and Chantrachit, 2001; Jaroenkit and Paull, 2003; Reis et al., 2020a and Reis et al., 2020b; Malakar et al., 2022). The main pros of tropical flowers are the eco-friendliness characteristics as mentioned by Darras (2021) while the current global floral trends i.e ‘Sustainable’ and ‘Wellbeing’ (Kaishita, 2022) also endorse the significance of its cultivation. These could reinforce their growing as mentioned by several researchers (cited herein). Hence, the consistent production of tropical flowers under tropical and sub-tropical regions not only foster the aesthetic and environmental significance but could also revive the tradition of their worthwhile usages with remunerative potential eulogistically. However, tropical flowers represent only approximately 4%-5% of all cut flowers traded (Laws, 1998 and Laws, 2005).

Tropical flowers consist of a diversified group of flowers that are native to tropical and subtropical climates (Paiva and Beckmann-Cavalcante, 2023). The most commonly known tropical flowers in the markets are cut orchids (*Cymbidium*) and *Anthurium*, but this product group also includes many other species belonging to the monocotyledonous taxonomic order of Zingiberales (Scitaminae, Clade - Commelinids) (Kress, 1990). This order includes eight families: Musaceae, Strelitziaceae, Lowiaceae, Heliconiaceae, Zingiberaceae, Costaceae, Cannaceae, and Marantaceae.The respective genus and species of these families usually grow in tropical regions (Cronquist, 1978).

A recent classification, elaborated by International Association of Horticultural Producers (AIPH) (2019), describes the production and consumption markets at global level, divided into four homogenous geographical groups: (1) Mature domestic producer countries (Europe, Canada, US, China, and Japan); (2) Emerging domestic producer countries (India, Mexico, and Brazil); (3) Mature exporting producer countries (Colombia, Kenya, and Ecuador); (4) Emerging exporting producer countries (Ethiopia and Vietnam). Producers and exporters of tropical cut flowers are present in these different groups, although it is often difficult to obtain separate market statistics for each product (Silva et al., 2019).

Mexico, Colombia, Ecuador, and Costa Rica are also considered as major exporters to USA and Canada, thanks to the benefits of NAFTAAlliance(North American Free Trade Agreement). Tropical flowers represent a relatively small segment of the European flower market and are traded at flower auctions in Holland (Royal Flora Holland) and Germany (Veiling Rhein-Maas), where a small number of merchants specialize in tropical flowers. However, important companies like Dutch Flower Group BV ([e-source: https://dfg.nl/en/] and Dümmen Orange [e-source: https://na.dummenorange.com/site/en] are involved in this market. The most commonly known tropical flowers in European markets are cut orchids (*Cymbidium*) and *Anthurium*. The product group also includes many other species, such as *Strelitzia*, *Heliconia*, *Protea*, *Leucadendron*, *Leucospermum*, *Ananas*, *Calathea* and Zingiberales (*Ginger*).In the South-East Asian province, China is the largest producer and exporter of tropical cut ornamentals. *Anthurium*, *Dahlia*, Lily, orchids and *Zantedeschia* (Calla Lilly) are the major tropical cut flowers exported to Dutch flower auctions from this province. Europe (mainly Germany and Italy), Japan and US are the largest importers of tropical flowers (Laws, 1998; COLEACP (Europe-Africa-Caribbean-Pacific Liaison Committee), 2002; Pizano, 2005; Linares-Gabriel et al., 2020; Chandel et al., 2022; Nzomoi et al., 2022).

In Europe, tropical flowers are primarily sold in mixed and colorful bouquets. Consumers appreciate these exclusive tropical bouquets and are willing to pay extra for them.In the Netherlands, a tropical bouquet consisting of*Anthurium*, *Heliconia*, *Celosia* and *Ananas*can cost around € 40/50 at the florist shop. In supermarkets, a single tropical flower, such as a smaller *Strelitzia* or Protea, is usually combined with cheaper flowers and fillers, and a small bouquet may cost as little as € 5 (CBI Product Factsheet, 2016). Recent consumption trends, marketing strategies, and governance settings in ornamental horticulture have been elucidated by Gabellini and Scaramuzzi (2022), but no mention of tropical ornamentals has been found.

According to the report of CBI Market Intelligence (2016) on the ‘Tailored study of tropical flowers and foliagefrom Colombia, UAE, and other Middle East markets’, *Heliconia* and gingers are of great importance. Both of these flowers originate from Asian countries such as Thailand and Malaysia. However, the main producing and exporting countries of tropical ornamentals of the Zingiberalesorder are Colombia and Costa Rica. Although no quantitative data related to the export, import, and detailed statistics on national and international trade of *Heliconia*have been found through bibliographic searches, it is reported that Brazil (54.5%), Colombia (15.4%) and Mexico (10.9%) are at the forefront of its production (Linares-Gabriel et al., 2020). India has been identified as the emerging domestic producer of this Specialty Tropical Cut Flower (STCF) among the Southeast Asian countries (Malakar et al., 2022).The price of a cut stem is approximatelyUS$2-3 or US$3-5 for the erected (80-100 cm length) or the pendent helicônias (e.g.*H. rostrata*)respectively (e-Source: https://www.cbi.eu/events/royal-floraholland-trade-fair-rfhtf-2016; no recent data are available).

Taking into consideration the great potential and burgeoning demand as a cut flower, our review will focus on *Heliconia*(>250 inter and intra-specific varieties are existing), commonly known as ‘False Bird-of-Paradise’, which belongs to the family Heliconiaceae(Abalo and Morales, 1982; Kress, 1990; Berry and Kress, 1991; Criley and Broschat, 1992; Abadie and Berreta, 2001; Urooj-UI-Nissa et al., 2015; Malakar et al., 2016; Avendaño-Arrazate et al., 2017; Krause et al., 2023). This review will specifically focus on the pre- and post-harvest factors that affect the desired attributes of heliconia cut flowers as expected by consumers. To the best of our knowledge, there is currently no dedicated review exclusively focused on post-harvest practices in Heliconia.

The state-of-the-art of scientific investigations on Heliconia reveals that research is mainly carried out in Brazil (67%), Colombia (19%),with a focus on production-related aspects (75%) rather than post-harvest (16%), marketing (7%), and industry-based (2%) research areas(Linares-Gabriel et al., 2020). On the contrary, scientific literature on post-harvest management practices for common tropical ornamentals (*Strelitzia*, *Anthurium*, Ornamental gingers, *Cymbidium, Dendrobium* etc.) is available and accessible. Therefore, in this review, we have considered the available literature on post-harvest practices in Heliconia, but we have also provided a brief overview of other related tropical members to present a comparative view of advancements in post-harvest measures and to support certain concepts. On the other hand, Jaroenkit and Paull (2003) stated that post-harvest management practices in all tropical ornamentals are more or less similar, possibly due to the structural anatomical similarities in peduncles (for inflorescences) or pedicels (for flowers). However, a detailed literature survey challenges the retention of this view.

In the following sections, we will take into account the factors that could affect the quality of Heliconia cut flowers throughout the entire production chain, from 'field to consumer.' This includes pre-harvest factors such as agronomic practices (Criley and Paull, 1993; De et al., 2014), harvest factors as harvesting maturity and seasons (Pompodakis et al., 2005) and post-harvest factors such as chain management and post-harvest handling sequences (Dolan and Sutherland, 2002; Collins and Dent, 2022). In a meta-analysis study on tropical plant postharvest, it was identified that the addition of preservatives to solutions, variations in storage temperature, and the use of electromagnetic fields are, currently, the most effective techniques in extending the shelf life of cut flowers (Cunha Neto et al., 2023). We will also consider post-harvest physiology determinants, including multiple genetic factors, maintenance of water balance components, and respiratory substrates (Onozaki et al., 2001; Fanourakis et al., 2013).

In summary, this review will highlight the challenges faced and effective measures to enhance the growing impact of tropical Specialty Cut Flowers (SCFs), along with providing directional hypothetical views on the necessary advancement for future perspective.

## 2 Post-harvest quality in *Heliconia*

Flowers are ephemeral organs (Smyth, 2005; Costa et al., 2021) due to their highly perishable nature, which results in a short post-harvest life. The quality of the final products plays a crucial role in determining the acceptability of cut flowers. Ensuring marketable post-harvest quality, which is a basic requirement for different market channels, necessitates maintenance at both pre- and post-harvest stages. Generally, the factors that affect the flowers physiology are classified as ‘Pre’ (including all aspects of agronomic practices) (Criley and Paull, 1993) and ‘Post-harvest’(abiotic factors such as storage temperature, relative humidity [RH], atmospheric gas composition, and biotic factors such as microbial occlusion and deterioration of physiological mechanisms) (Fernandes et al., 2020; Costa et al., 2021). Harvesting (considering maturity and the season/time of cutting) and handling (including sorting, storage, packing, and transport) of harvested produce are also vital factors to consider.

Among the most common drawbacks that can affect post-harvest quality, there are several factors that apply to various tropical species. These factors include the lack of standardization of harvest indices, inadequate handling facilities, negligence during handling, sharing of storage space with other traditional cut flowers, and more. Additionally, structural differences in floral taxonomy, sensitivity to chilling, large size, special packaging requirements, and anatomical specifications can also contribute to these challenges. Due to these factors, post-harvest practices cannot be generalized, and special attention should be given to the specific species' post-harvest requirements (Jaroenkit and Paull, 2003).

In *Heliconia*, its inflorescences (bold cincinnus type; with either an upright or pendant posture) consisting of multihued bracteate structures with spiral or distichous aestivation.) (Kress, 1984; Criley and Broschat, 1992; Criley and Paull, 1993; Castro et al., 2007; Rodríguez, 2013; Costa et al., 2015; Loges et al., 2016; Souza et al., 2016). These inflorescences are popularly used as cut flowers, unlike its true flowers, which are inconspicuous in nature, usually white, yellow or orange in color, numbering from 2 to 20 and remaining enveloped by bracts (Criley and Paull, 1993, Criley and Broschat, 1992, Krause et al., 2023). The importance of Heliconia as a cut flower lies in the bright hues, sturdiness, and freshness of its inflorescences. However, ill-developed or deteriorated chromatic features and desiccated or browning symptoms of the boat-shaped bract tips categorize them as non-acceptable cut units. To overcome these drawbacks, the role of several factors mentioned in the preceding paragraph will be delineated in the following sections.

### 2.1 Pre-harvest factors

Very few research reports have been obtained on the role of pre-harvest factors in determining the post-harvest quality of cut *Heliconia*. Similarly, when considering other allied cut tropical ornamentals such as *Strelitzia* sp. and ornamental gingers (OGs), apart from Anthurium, the status of research investigations on pre-harvest factors as indicators of after-harvest quality of cut flowers is very limited. Nonetheless, achieving a high yield of quality cut units is the main objective of manipulating pre-harvest factors. In this section, the roles of light, temperature, and fertilizers as optimum standards for boosting yield and determining post-harvest quality will be discussed.

**Light:** Optimum light condition impacts on the phenotypic features of plants, hence, it is considered as one of the crucial factors during the agronomic practices. Concerning tropical ornamentals, generally, the light requirement varies from genotype to genotype. According to Broschat and Donselman, 1983a; Broschat and Donselman, 1983b) *Heliconia* can thrive best under the semi-shaded condition while excessive shade and crowding can cause the reduced yield with weak, lanky stems as most evident in *H. psittacorum* groups. Malakar et al. (2016) had also corroborated the same based on the outcomes of their experiment on evaluation of agro-morphological attributes of diversified *Heliconia* genotypes under West Bengal, India, condition but any standardized range of shade percentage yet to be made. The other tropical cut flowers also prefer semi-shaded condition like in *Alpinia* 30% (Criley, 1988), *Curcuma* 50-70% (Criley, 2014), *Anthurium* 30%-75% (Kamemoto, 1962) while according to Ismail et al. (2019), *Etlingera* needs partial shade (standardized percentage not found) but the empirical views of the authors of this review are paradoxical to the view of these researchers.

The flowering in *Heliconia* is seasonal and depends on certain growth condition and physiognomic features (eg. in *H. angusta*, pseudostem bearing 3 unfurled leaf blades can only cause bud emergence, in *H. chartacea*, number of emerged shoots and leaves determine the onset flowering) as well as place of growing (Criley and Lekawatana (1994) but a few research reports on roles of photoperiod for growth and flowering have been noticed. According to Criley et al. (1999), the photoperiodic requirement, for flowering in *Heliconia* genotypes, is species-specific. They found *H*. *Wagneriana* and *H*. *stricta* var. 'Dwarf Jamaican' as short day (SD) species while *H*. *angusta* as long day (LD). They also mentioned that in *H. angusta*, if exposure to LD condition for>13.3 h for the duration of 15 to 17 weeks takes place, then only the anthesis can happen while in rest 2 species, anthesis takes place after 15 to 19 weeks of exposure to SD condition. Again Geertsen (1990) found that to get early, abundant flowering along with shortened peduncle length in *H. aurantiaca* Ghiesbr. ex. Lemaire, exposure at 16h of LD condition is optimum while to get off-season blooming in *H.rostrata*, critical day length condition of 11.5-12h; in other words, SD condition, is needed (Maciel, 2000). The photoperiodic requirement of other tropical ornamentals is basically LD while some exceptions also could be found (**Table 1**).

**Table 1:** Pre-harvest photoperiodic requirement in some tropical cut ornamentals.

Names of genotypes	Common names	Photoperiodic requirement	Remarks	Sources
*Alpinia* sp.	Red ginger	Long day (LD)	–	Criley and Maciel, 2002; Criley, 2014
*Hedychium* sp.	White ginger lily	Long day	–	Agboka and Criley, 2002; Changjeraja et al., 2008
*Curcuma* sp.	Curcuma	Long day (>13h) either by night interruption lighting or day length extension	–	Hagiladi et al., 1997; Sarmiento and Kuehny, 2003; Criley, 2013 and Criley, 2014)
*Etlingera* sp.	Torch ginger	Short day (SD)	Based on the practical experience, it may be said that it is LD photoperiodic plant because a few of the places in Brazil where high temperature and LD condition prevails perpetually, there round-the-year flowering can be experienced while in South-Eastern part of Brazil where low temperature (average 10-15°C) as well as SD conditions occur during winter season, flowering found absent. In nutshell, the influence of temperature factor rather than the light factor is more pronounced.	Criley, 2011 and Criley, 2014, Castro et al., 2011
*Strelitzia* sp.	Bird-of-Paradise	Photoperiod insensitive	–	Halevy et al., 1976
*Anthurium* sp.		LD (16h)	–	Budiarto, 2010

-, No information.

**Temperature:** Concerning *Heliconia* cultivation, like light intensity, temperature is also another major factor but dearth of scientific investigations in this regard have been evidenced; hence, only a handful of old scientific reports have been cited here. According to Broschat and Donselman (1987), the air temperature below 10-12.5°C could be harmful for *H. psittacorum* while harvesting season also matters as reported by Bredmose (1986). Both the researchers mentioned about the vase life (V) difference in *Heliconia* genotypes grown in Denmark due to different harvesting season (winter harvesting V_short_ and summer harvesting V_long_). Again Geertsen (1990) had been found to be reported that the temperature range between 15 to 21°C could cause increase in flowering percentage by >20% along with the peduncle length of 40 cm while the number of leaves subtending the inflorescence was increased by 2.5% in *H. psittacorum*. Any report on frost sensitiveness of *Heliconia* has yet be found while the impact of exposing its inflorescence under varied degree of light intensity is also absent. The state-of-the-art on this aspect regarding other tropical ornamentals like *Alpinia*, Anthurium, Strelitzia, Curcuma etc. are also unsatisfactory (**Table 2**); apparently proves the research negligence on current date, which embody the importance of conductance of research investigation on them including *Heliconia*.

**Table 2:** Pre-harvest temperature requirement in some tropical cut ornamentals. .

Names of genotypes	Temperature requirement	Impacts	Remarks	Sources
*Alpinia* sp.	<16 and >10°C^1^; 21°C^2^	^1^Optimum for high yield; ^2^Early emergence of flowering stalk and diminish of frost sensitiveness but exposure to temperature >25^0^C cause the onset of ‘Tip burning disorder’.	The empirical views of authors of this review cannot fully endorse these results.	^1^ Criley, 1984 and Criley, 1988; ^2^ Criley, 1988
*Curcuma* sp.	Tropical temperature (temperature specifications did not find)	Seasonal blooming	To have perpetual blooming, forcing technique which involves the low temperature (15°C) storage of rhizomes followed by warmer storage (25-32°C) for 15days, found effective.	Criley et al., 2003; Sarmiento and Kuehny, 2003; Paz et al., 2005; Roh et al., 2005; Criley, 2014
*Anthurium* sp.	<25°C during day and 18°C during night	Improved postharvest quality	Temperature should be critically maintained since temperature and deterioration in post-harvest quality of it are inversely co-related with each other	Paull, 1991
*Strelitzia* sp.	–	–	Standardized threshold range is yet to determine but PGRs, especially GA, plays significant role to reduce its temperature sensitivity; cause increased length and number of emerged flowering stalk along with early inflorescences’ maturation during off-season cultivation.	Halevy et al. (1976), Criley (2015)

-, No information.

**Fertilizer:** In 1987, Broschat and Donselman worked on the effect of NPK on qualitative and quantitative evaluation of cut stems of *H. psittacorum* and found that K does not have any impact while N up to 600gm^-2^ could augment the yield and quality attributes both. Recently, Sardinha et al. (2019) had carried out an investigation on the influence of phyto-stimulants (like Bion, Agro-MOS Quartz, Ca-Fosfitotal (Ca-Phosphite), K-Fosfitotal (K-Phosphite)), applied in the field condition, on the stem quality of *H. psittacorum* cv. ‘Golden Torch’ during vase life. They found the positive implications of them while the best outcomes had been obtained in K-Fosfitotal since electrolyte leakage (EL) and lipid peroxidation scores were low in this case. Other associated post-harvest parameters like visual aspect, fresh matter loss, water balance in cut stems were improved too by due application of phyto-stimulants during cultivation phase as pre-harvest management strategy (Sardinha et al., 2019).

Loss of cell membrane integrity generally is triggered by electrolyte’s leakage which consequently causes the cell death. (Hatsugai and Katagiri, 2018). Electrolytes like Ca^2+^, K^+^, Mg, Chlorite, Ph, Na^2+^ etc. play vital role in determining the post-harvest physiology of cut floral units. High concentration of electrolytes aids to withstand various abiotic and biotic post-harvest stresses (like water stress condition, pathogen attack etc.) while its low quantity causes the reverse (Hatsugai and Katagiri, 2018). On other side, peroxidation of lipids can disturb the assembly of the membrane by causing changes in fluidity and permeability, alter the ion transport and inhibition of metabolic processes. High scores of it, cause injured mitochondria inducing higher rate of ROS generation (Pourzarnegar et al., 2020) and consequently affect the post-harvest phase of ornamental cut units. Different enzymatic activities like peroxidase (POD), polyphenol oxidase, and superoxide dismutase (SOD) had been also influenced by application of phyto-stimulants (Sardinha et al., 2019). Optimum agronomic practices reduce the post-harvest oxidative stress by increasing anti-oxidant enzyme i.e. SOD and catalase (CAT) activities as reported by Zulfiqar et al. (2022).

Based on the bibliographic searches, it may be said that the nutritional requirement and its impact on post-harvest quality vary in different tropical cut flowers. The curated information(s) have been tabulated (**Table 3**). Furthermore, bract’s hue determines the value of tropical flowers; hence, the impact of fertilizers on maintaining the bract color is another vital aspect of investigation. In *Heliconia* and any other tropical ornamentals except Anthurium (**Table 3**), any such reports have not been identified.

**Table 3:** Status of research on pre-harvest nutritional requirement in some tropical cut ornamentals.

Names of genotypes	Fertilizer dose rates	Impacts	Remarks	Sources
*Strelitzia* sp.	–	–	Varied dose rates owing to the adoption of region-specific diverse cultural practices	Criley, 2014 and Criley, 2005
*Anthurium* sp.	N:P:K: 312:448:375	Quantitative (high yield) and qualitative (increased peduncle length) improvement;	Moderate to high level of K can improve the quality of spathe; P has no effect; high level of N impairs the quality but the combination of N and K, at high dose rate (rate did not found), cause the linear increase in spathe size may be due to the synergistic interaction effect;	Higaki et al. (1992); Paull (1991); Criley and Paull (1993)
*Anthurium* var. ‘Kamuna’ & ‘Ozaki’	Osmocote: 13.5:13.5:13.5	Alleviation of ‘Spathe bleaching disorder’	Temperature range should be 21-26°C for 9 weeks duration cause the optimum release of NH_4_-N and NO_3_-N	Mills, 1981; Leonhardt and Woomer, 1991
*Alpinia* sp.	N:P:K level ranges from 1:1:1 to 3:1:5 [under Florida condition] while 300kg N ha^-1^[under Venezuela condition])	Optimum yield	Place of growing affect the dose rates.	González and Mogollón, 2001; Criley, 2014 and Criley, 1988
*Etlingera*sp.	NPK: 200g plant^-1^; organic compost of 1kg month^-1^;	Improved quantitative and qualitative attributes	–	Criley and Maciel, 2002; Araújo et al., 2018,
*Hedychiumcoronarium*	NPK: 19:19:19 along with garlic extract	Improved flowering attributes like main stalk length and diameter, inflorescence number clump^-1^, rachis length, florets number inflorescence^-1^ and fresh and dry weight of flowering spikes	–	Attia et al., 2020
*Curcuma* sp.	N:K: 200 ppm each	Improved quantitative and qualitative attributes	–	Ruamrungsri et al., 2006
*C. alismatifolia* x *C. cordata* cv. ‘Laddawan’	N:P:K: 20:20:20 combining with chitosan: 20mgL^-1^	Improved post-harvest quality	Foliar spray application	Uthairatanakij et al. (2007)

-, No information.

**Other factors:** Any scientific reports on standardized spacing, irrigation and plant growth regulators (PGRs) regime for *Heliconia* cut flower production have not yet found. But in other tropical cut flowers, meager information(s) have been obtained. As for example, for *Alpinia* and *Etlingera*, optimum plant-to-plant (P/P) spacing are 1.2-2.0m (Criley, 1988), and 1.5 x 1.5m (Criley, 2011 and Criley, 2014, Castro et al., 2011) respectively while irrigation of 25mm week^-1^in *Alpinia*(Criley, 2014) and withholding of irrigation in scheduled manner for about 2.0-2.5 months, had been found beneficial in early flowering in *Strelitzia* (Criley, 2005). In case of *C. alismatifolia*, *C. gracillima* and *C. thorelli*, pre-soaking of rhizomes in GA_4+7_solution (concentration did not found), for 10mins duration, during pre-planting stage may cause the dwarfing of floral stem length (Sarmiento and Kuehny, 2003) which consequently make these cut blooms more feasible to use and transport also. Again, according to Hagiladi et al. (1997), the planting of propagules with >5 t-roots (tuberous egg-shaped root ends of rhizome) as compare to<2 t-roots, result in early flowering in ornamental *Curcuma*.

Surprising augmentation in demand of tropical flowers in international floral market has alarmed to be mindful about the quality and phyto-sanitary aspects of cut flowers. Several fungal causative agents like *Calonectria spathiphylli* (susceptible genotypes: *H. angusta* cv. ‘Holiday’, *H. bihai* cv. ‘Lobster Claw’), *Bipolaris incurvata* (susceptible genotypes: *H. stricta* var. ‘Dwarf Jamaican Red’, *H. chartacea*), *Cercospora* sp. and *Pseudocercospora* sp. (susceptible genotypes: *H. psittacorum* cv. ‘Andromeda’, *H. wagneriana*) cause severe foliage diseases while *Rhizoctonia solani* (susceptible genotypes: *H. bihai* cv. ‘Lobster Claw’, *H. caribaea*), *Phytophthora nicotianae* (susceptible genotype(s): *H. caribaea* and highly tolerant genotype(s): *H. mutisiana*), *Pythium* sp.(susceptible genotypes: *H. psittacorum* var. ‘Bengal’, *H. indica* cv. ‘Spectabilis) - these fungal strains found responsible for the rhizome and root diseases (Ferreira et al., 1991 and Sewake and Uchida (year missing, e-source: http://www.extento.hawaii.edu/kbase/reports/heliconia_pest.htm). Barring these, several bacterial diseases like ‘Leaf Rolling’, ‘Wilting’ and ‘Die back’ also found to be caused by *Pseudomonas solanacearum*; *H. psittacorum* and *H. rostrata* show the high susceptibility (Ferreira et al., 1991) towards it while ‘burrowing’ nematode (*Radopholus similis*), ‘root-knot’ nematode (*Meloidogyne* sp.), and ‘lesion’ nematode (*Pratelenchus* sp.) have also been reported to infect *H. angusta, H. chartacea, H. stricta* (Sewakeand Uchida (year missing, e-source: http://www.extento.hawaii.edu/kbase/reports/heliconia_pest.htm). Any such viral diseases have yet to be reported in *Heliconia* but Hamim et al. (2017) had reported about the occurrence of BBTV (Banana Bunchy Top Virus), for the first time ever, in *H. aurantiaca* in Hawaii. Positive result of triple-antibody sandwich (TAS)-ELISA and PCR tests had confirmed the infestation but any remedial measures had not been stated by the researchers. In case of *Anthurium, Xanthomonas* bacterial blight led lesions on spathe often impairs the quality of cut units (Norman and Ali, year missing; e-source: https://edis.ifas.ufl.edu/publication/PP292; Hara et al., 2004).

Generally, several pre-harvest management practices determine the occurrence and spreading of diseases; Heliconia is also not exceptional. The mode of spreading is significantly diverse; as for example, may be through soil contamination, contact infestation, water stagnation condition (synonymously poor drainage), influence of various abiotic factors etc. In most cases, ‘rhizome rotting’, caused by bacterial and nematodal infestations; mainly spread from soil to roots, results in dying of Heliconia plants while the degree of foliage’s infestation also directly affects the plant health and consequences into low yield with impaired quality’s inflorescences (Berry and Kress, 1991).

To date, barring the conventional measures viz. optimum cleaning of rhizomes, controlled soil moisture level, appropriate spacing between plants for good air movement, sanitation of growing substrates, enhancement of soil organic matter content, use of resistant genotypes, application of insecticides and pesticides, soil fumigation processes, any modern approaches to mitigate the said menace have not been devised concerning our discussed ornamental’s group. El-Baky and Amara (2021) mentioned about some novel advanced approaches towards controlling phytopathogenic issues. Several strategies like biofumigation (employs organic material’s fermentation to develop anaerobic condition and toxic metabolites to make phytopathogens non-functional), use of antagonistic microorganisms in virgin soils, use of microbial fungicides by unique application method via honey bees, known as ‘flying doctors’, as they deliver bacterial fungicide *Bacillus subtilis* during pollination, use of agronanotechnology. Fungal cell deactivation and evacuation using ‘Ghost’ technique, use of UV light and floral extracts like Chammomile tea and modern breeding strategies like RNA interference (RNAi) (i.e host-induced gene silencing [HIGS] and spray-induced gene silencing [SIGS]) to develop phytopathogenic resistant plants have been mentioned by them which are based on ‘Green principles’ and are already in practice in case of other horticultural crops. However, the conductance of trial tests of these said techniques aiming to minimize not only the occurrence of phytopathogenic interference but also to produce sustainably export quality’s cut inflorescences. A few tropical flowers importing countries after disembarkation of the cut produces, clean (either by simple hot water treatment or by foamy soap water cleaning) them on-site (Berry and Kress, 1991) rather following any stringent phytosanitary regulations.

Summarily, it may be said that the optimum field management practices could necessarily ascertain the qualitative and quantitative features of post-harvest products. So, resorting to afore cited reports, some ventures might would be also taken in *Heliconia* for further future investigations.

### 2.2 Harvesting factors

Generally, *Heliconia* inflorescences are harvested by cutting near the stalks (Criley and Paull, 1993; Criley, 1995) while on the contrary, Broschat and Donselman (1983b) found satisfactory keeping quality with the pulling method of harvesting. Harvesting time (in terms of season and hours) and stage of harvesting are key factors determining the post-harvest quality of cut flowers. Generally, bract opening after harvesting does not occur in *Heliconia* inflorescences (Criley, 1995). To achieve the best post-harvest quality, harvesting cut units from well-irrigated plants could be a way to reduces the chance of desiccation to some extent (Dolselman and Broschat, 1986). Harvesting indices are usually genus and species-specific. For large-sized *Heliconia* inflorescences, harvesting at the stage of ½ or 2/3^rd^ open bracts, while for a few 1 small-sized cultivars of *H. psittacorum*, harvesting at the stage of 1 or 2 open bracts or tight bud stage, has been found to yield optimum shelf life and post-harvest performance, as stated by Broschat and Donselman (1983b) and Criley (1995). In Strelitzia sp. and *Alpinia* sp., the harvesting indices are almost similar, while in *Etlingera*, ‘tight bud stage’ and ‘torch stage’ are considered as the optimum stage of harvesting (Criley, 2014), although it may vary from species to species (**Table 4**). According to Liju (2013), harvesting the inflorescences at the 1-2 open bracts stage in general increases the vase life. Tjia and Sheehan (1984) stated that harvesting immature inflorescences could extend the keeping quality by an extra 42% compared to mature harvesting (3-4 bracts open stage). The retention of foliage in harvested inflorescences generally does not affect their post-harvest life (Ka-ipo et al., 1989; Criley and Broschat, 1992; Criley, 1996). Generally, for large and small types of *Heliconia* (eg.*H. psittacorum*’s cultivars), a total stem length of 150 cm and 60-90cm, is maintained (Criley and Paull, 1993), while for *Alpinia* and Etlingera, peduncle length of 60-150cm (Criley and Paull, 1993) and 50-70cm (Araújo et al., 2018; Baskaran, 2022), respectively, are maintained. The standardization of maturity threshold for harvesting allied tropical ornamentals like *Globba*, *Curcuma*, and *Zingiber* species is yet to be made.

**Table 4:** Harvesting indices of some tropical ornamentals.

Names of genotypes	Optimum harvesting condition/indices	Sources
*Strelitzia* sp.	Tight bud condition (orange colored tepals start to peep from the spathe)	Criley and Paull, 1993; Jaroenkit and Paull, 2003; Bayogan et al., 2008; Koley, 2013; Criley, 2014
*Alpinia*sp.	½ to 2/3^rd^ or 3/4^th^ number of open bracts with 2 or 3 attached foliage(s)	Broschat and Donselman, 1988; Criley, 1996 and Criley, 2014
*Etlingeraelatior*	Semi-opened inflorescence [initiation of unfurling of basal bracts and visible central portion of inflorescence]	Castro et al., 2013; Hintze, 2013, and Hintze, 2014
*E. haemispherica*	Do	Do
*E. corneri*	Tight bud stage	Do
*Hedychium*	Shape and arrangement of bracts of cone to terminal’ and ‘tight to loose’ respectively	Wood, 1999; Criley, 2014

"Do" refers to same as above.

Several physiological reasons lie behind harvesting at the optimum maturity stage, but there is no evidence of any investigations concerning this in *Heliconia.* To support this, it may be mentioned that the activities of cellulase and pectin methyl esterase enzymes, which principally regulate flowering and post-harvest abscission, respectively, are determined by the optimum harvesting stage, as evidenced in *Etlingera* by Wang (2017). High cellulase activity has been found in involucral bracts of *Etlingera* from the ‘tight bud’ to the ‘6-tip opened stage’, but its activity deteriorates from ‘6-tip opened stage’ to the ‘torch stage’, while the activity of pectin methyl esterase increase (Yeat, 2016). The same researcher reported that from the ‘tight bud’ to the ‘bloom stage’, a high content of ethanol insoluble residue and cellulose content in the peduncle are usually found. Taken together, it may be said that further insight into the physiological basis of determining the harvesting stage of tropical cut flowers may be the timely need.

### 2.3 Post-harvest factors

The *Heliconia*, as a cut flower, is highly accepted by customers due to its diversity in bract color and exotic appearance (Loges et al., 2005 and Costa et al., 2011; Loges et al., 2016). Therefore, preserving the hue of its bracts is one of the primary goals during their presence with consumers. The hardy nature, firmness of the peduncle, and natural durability (Castro et al., 2006, Reid, 2001) can also be considered as additional features that contribute to their high demand. However, to maintain the post-harvest quality of cut units, several factors need to be carefully taken into account. Temperature, water balance, carbohydrate supply, and growth regulators (Reid and Kofranek, 1980; Halevy and Mayak, 1981) are key factors that play a crucial role in the storage of cut items and in extending their vase life. In the following sections, we will discuss the role of each factor in the post-harvest vase life of *Heliconia* cut inflorescences, as well as other related tropical cut flowers.

**Temperature:** The recommended storage temperature for cut *Heliconia* is >12°C (Broschat and Donselman, 1983b; Criley and Paull, 1993) since all species and cultivars of this genus are highly sensitive to low temperature condition. chilling injury (CI) such as depressions, browning, or dark spots on the bracts and flowers can occur if they are stored at temperatures below 10°C for 2 days, as reported by several researchers (Markhart, 1986; Paull, 1991; Darras, 2020 and Carrera-Alvarado et al., 2021). Silva et al. (2019) evaluated the optimum storage temperature of *H. densiflora* and *H. psittacorum* (in three different color varieties: ‘Red’ [5R 4/10], ‘Orange’ [7.5Y 7/10], and ‘Yellow’ [2.5Y 7/10]) under controlled temperature condition. The samples were stored at three different temperatures (14°C, 18°C, and 22°C) inside a cold chamber and under controlled ambient conditions (26°C). They found that 14°C (storage period up to 9 days) was optimal for the ‘Red’ variety, and 14-22°C (for a storage period of up to 6 days) was optimal for both the ‘Yellow’ and ‘Orange’ varieties of *H. psittacorum*, with no signs of senescence or necrosis. For *H. densiflora*, a temperature of 18°C was recommended for storing healthy cut stems for up to 6 days. Controlled ambient conditions were found to be unsuitable for storage (maximum storage period recorded was 1-2 days). Costa et al. (2011) also studied the storage of *H. bihai* cv. ‘Lobster Claw’ at low temperatures (12°C and 19°C). The development of CI symptoms was observed from the 3rd day onwards when stored at 12°C, while storage at 19°C for up to 8 days was recommended for maintaining visual quality and fresh weight of the cultivar.

CI is a physiological malfunction of plants caused by exposure low temperatures rather than freezing temperatures, rendering the product unusable (Markhart, 1986; Parkin et al., 1989). Loss of membrane integrity due to low-temperature-induced membrane lipid phase transitions and physiological dysfunctions are the fundamental biochemical causes of CI (Bhattacharya and Bhattacharya, 2022). The high concentration of high melting phospholipids, membrane retailoring, increased fluidity at low temperatures, direct or indirect impact on intrinsic enzymes due to membrane perturbations, and redistribution of cellular Ca (which acts as a secondary messenger of many cellular functions) may also be considered as primary transducer of CI (Parkin et al., 1989; Chen and Ko, 2021; Tian et al., 2022; Olmedo et al., 2023). Maintenance of membrane integrity enables the electron transport chain system and oxidative phosphorylation to produce ATP and supports the normal functioning of pathways like TCA cycle, glycolysis, β-oxidation, etc (Parkin et al., 1989; Darras, 2020), but disruption of membrane structure impacts the entire physiology. According to Lukatkin et al. (2012), cyto-physiological changes such as distorted cell membranes leading to loss of cell compartmentation, swelling and rupture of plasmalemma, destruction of the endoplasmic reticulum and vesiculation of its membranes, changes in the Golgi apparatus, swelling and degeneration of mitochondrial structure, matrix enlightenment, and cristae shortening also occur due to CI. CI also affects photosynthesis, as reported by Markhart (1986) and Lukatkin et al. (2012). They mentioned that chilling-induced water stress decrease the quantum yield of whole-plant photosynthesis and also cause direct injury to the chloroplast (the oxidative side of photosystem II is the site of injury). Furthermore, decreased mitotic cell index (Strauss et al., 2007), cessation of cell growth (Rymen et al., 2017), decreased cytoplasm viscosity (Lukatkin et al., 2012), coagulation of structural proteins (Zhang et al., 2021), low soluble protein content, shift in intracellular pH (Kasamo et al., 2000), cessation of cytoplasmic streaming (Lukatkin et al., 2012), and changes in ATP (adenosine-5’-triphosphate) levels (Minorsky, 1985) are also part of the implications. Parkin et al. (1989) hinted at the role of lipid peroxidation in causing irreversible damage, during low-temperature storage, in the form of free radical-induced damage to tissues and progressive membrane rigidification.

Generally, all tropical ornamentals except *Strelitzia* (**Table 5**) are susceptible to low-temperature storage. In this context, *Anthurium* should be mentioned first (Promyou et al., 2012). The key CI symptoms of *Anthurium*are ‘Browning’ and ‘Blueing’ of the spathes and wilting of spadixes (Promyou et al., 2012; Aghdam et al., 2015). Generally, storing Anthurium at an optimum temperature of 10-12°C controls these symptoms (Reid and Wu, 1992; Balas et al., 2006; Aliniaeifard et al., 2020). However, Paull (1987) found exceptions regarding the storage of some other Anthurium varieties at higher temperatures (14-17°C). Chemical solutions, such as salicylic acid and GABA-based treatments, have also been shown to alleviate CI issues (Promyou et al., 2012, Soleimani Aghdam et al., 2015 and Aghdam et al., 2016). However, none of these strategies completely eliminate browning of the spathe. Recently, Aliniaeifard et al. (2020) conducted an experiment to evaluate the role of different post-harvest light spectral compositions (Red [R], Blue [B], R & B@ 70:30% and White [W]; intensity - 125µmol m^-2^S^-1^) in alleviating chilling injury symptoms in ‘Calore’ (red-colored spathe) and ‘Angel’ (white-colored spathe). They observed that protection from blue light could reduce chilling injury, as indicated by lower electrolyte leakage, and water loss percentage during storage under blue light exposure (storage temperature was 4^0^C). They also found that the short vase life of the blue light-treated cultivar may be attributed to the effect of this light spectrum on oxidative stress and membrane integrity. Despite categorization Heliconia as a highly chilling-sensitive crop, no investigations on the role of light spectrum in alleviate this issue have taken place yet. However, a few investigations on this aspect have been conducted on other related tropical ornamentals, and the findings are summarized in **Table 5**.

**Table 5:** Research status on impact of post-harvest factors on qualitative attributes of some tropical ornamentals.

Names of genotypes	Purpose of evaluation(s)	Factors				Impacts	Remarks	Sources
		^A^Storage Temperature	^B^Water balance	^C^Preservative solutions				
				^C1^Chemical(s) based	^C2^GR based			
Strelitzia sp.	^A^Standardization of temperature to retain post-harvest quality; ^C1f^Efficacy of GO andAgNPs as anti-microbial agents & to extend the vase life longevity ^C1g^Efficacy of Ce(NO_3_)_3_ as pulsing and holding solution	>8^0^C^a^; ≤7-15^0^C^b^; 6-7^0^C^e^; 10-13^0^C^c^	–	^f^GOandAgNPs: 1µML^-1^(each) (holding treatments); ^g^Pulsing (24h) by 300µM of Ce(NO_3_)_3_ &holding solution using 300-600µM of Ce(NO_3_)_3_	–	^A^Confusional; ^C1f^Vase-life extended >6 days over control along with improved RWU and FW retention, reduced rate of microbial blockage, decreased rate of electrolyte leakage and better enzymatic activities (high SOD, POD and low MDA); ^C1g^Pulsing treatment caused longer longevity of 11.68 days along withincreasedwater uptake, dry matter content, & FW &reduced bacterial populations at the cut stem end and in vase solution too, utmost POD (0.147nmol g^-1^ FW) and catalase activities (1.02nmol g^-1^ FW) along with lowest MDA accumulation (0.09 nmol g^-1^ FW) had also been recorded.	^A^Little tolerant to low temperature condition; in fact, may be at 0^0^C temperature for short term (7days) without occurrence of chilling injury (CI) symptoms (i.e bract and sepal discoloration)^d^	^Aa^ Jaroenkit and Paull, 2003, ^a^ Nowak and Rudnicki, 1990, ^b^ Rudnicki et al., 1991, ^c^ Vieira et al., 2012; ^d^ Reid and Jiang, 2012; ^e^ Reid, 2004; ^C1f^ Thakur et al., (2022); Yan and Chen (2019) and Sharma et al., (2022) ^C1g^ Azarhoosh et al., (2021)
*Alpinia*	^A^Standardization of temperature to retain post-harvest quality; ^B^Any investigations yet to be conducted; ^C1^Vase life extension; ^C2kl^Impact of GRs alone & in combination with other chemicals to extend vase life;	>12^0^C temperature and >80% of RH	–	0.1% of ascorbic acid treatment (as vase solution)	^k^BA: 100mgL^-1^ (dipping or spray) ^l^Combination of 5% of sucrose + 3mgL^-1^ of BA + 200mgL^-1^ of 8-HQC (holding solution)	^A^Minimization of CI symptoms; ^C1^It had protracted the longevity for 11.60days over control (8 days); ^C2k^Extended the vase life of 11.60days over control (8 days); ^C2l^Caused optimum enzymatic activities with reduced oxidative stress	^B^ To minimize BD disorder owing to high post-harvest transpiration water loss, optimum balance b/w all water balance components seems vital.	^A^ Broschat and Donselman, 1988; Paull, 1991; **^B^** Criley, 2014; ^C1^ Morais et al., 2012; Islam et al., 2013; ^C2k^ Paull and Chantrachit, 2001; ^C2l^ Morais et al., 2012
*Etlingeraelatior*	^A^Any investigations yet to be conducted; ^C2hij^Impact of GRs in combination with other chemicals to extend vase life& to improve postharvest quality;	–	–	–	^h^1-MCP treatment (holding solution) (concentration did not mention); ^i^Combination of 8-HQ: 100mgL^-1^+ GA: 50mgL^-1^+ sucrose: 2% + BA: 50mgL^-1^(as holding solution); ^j^Pulsing (30 min) using 200mgL^-1^ of BA (under ambient temperature of 27^0^C, 72% RH)	^C2 hi^Both found beneficial to get the vase life of 23.63 days over control (18 days). ^C2j^Improved vase life by 18.7% (over the control 10-15days), reduced senescence symptoms *viz.* browning of bracts, loss in gloss and hues.	^A^Onset of BB disorder (CI symptom) takes place for low temperature storage, depletion of soluble sugar and cellulose level, along with high respiration rate; ^C2j^Peduncle length of 65cm had been maintained	^A^ Yeat, 2016; ^C2h^ Bayogan and Gratuito, 2013; ^C2i^ Chaudhari et al., 2016; ^C2j^ Choresca et al., 2019
*Costus*	^A^Standardization of temperature to retain post-harvest quality	5-7^0^C; 25^0^C (temperature) & 75-80% (RH)	–	–		^A^Protraction of longevity at low temperature, like the higher one, without emergence of any CI symptoms	^A^Confusing outcomes and paradoxical in terms of empirical views.	^A^ Criley, 2014,
*Curcuma*	^C2mnop^Efficacy of GRs on vase life extension	–	–	–	^m^1-MCP: 900ppb (pulsing); ^n^100ppm of GA_3_ (pulsing); ^o^Combination of 1.8% of both GA_4+7_ and BA (each 2mgL^-1^) (dipping for 15hrs) ^p^1µM of STS (pulsing)(anti-ethylene growth regulator)	^C2mnop^useful for vase life extension	–	^C2m^ Chutichudet et al., 2011; Bunya-atichartet al. (2004), and Criley (2014); ^C2n^ Kjonboon and Kanlayanarat, 2004; ^C2o^ Favero et al., 2017; ^C2p^ Chanasut, 2004
*Anthurium x ferrierense*	^B^Impact of refrigerated condition on water balance components & the subsequent vase life.	–	Best T:WU ranged from 0.86-1.04	–	–	^B^Optimum water balance & extended vase life of 30days.	^B^13 and 18^0^C temperatures (refrigerated conditions) found most appropriate than ambient temperature (28^0^C) for maintaining balanced T: WU ratio.	^B^ Sankat and Mujaffar (1994)
*A. andreanum*cv. ‘Spirit’, ‘Success’, and ‘Hondurus’	^B^Evaluation of the impact of RWC on eradication of SN disorder	–	Positive relation b/w high RWC and low intensity of SN	–	–	^B^Extended vase life & alleviation of SN disorder.	^A^Normal range of storage temperature of cut blooms is 14-17^0^C. ^B^Low ion leakage, intact membrane integrity and optimum hydraulic conductance in spathe had been found.	^B^ Farrell et al. (2012)
*Anthurium* var. ‘Ozaki’	^C1^Impact of ammonium salts on color retention during after harvest period	–		NH_4_NO_3_ (9 and 15meq. NH_4_^+^) and (NH_4_)_2_SO_4_(9meq. NO_3_ and NH_4_^+^) as pulsing solution	–	^C1^Retention of spathe’s hue (temporary effect)	–	^C1^ Leonhardt and Woomer, 1991

*T:WU, Transpiration & water uptake ratio; RWC, Relative Water Content; SN, Spadix Necrosis; BD, Bract Desiccation; BB, Bract Browning; GR, Growth Regulator; RWU, Relative water Uptake; FW, Fresh Weight;GO, Graphene oxide; AgNPs, Silver Nano Particles; MCP, Methyl cyclopropane; STS, Silver thiosulfate; MDA, Malondialdehyde -, No information.

Principally, the major post-harvest deteriorations occur during the shipment of the produces, and the temperature conditions at the destination also play a vital role in this case (Paull, 1991). Since our target crop originates from tropical and sub-tropical regions, when it is shipped to temperate regions, the sudden low-temperature shock poses a vulnerability. However, prolonged exposure of the cartons to scorching temperatures due to unprofessional handling can also cause injury (Reid and Kofranek, 1980; Heliconia Society International (HSI), 1989). Leite et al. (2015) evaluated the optimum storage conditions for *H. stricta* var. ‘Bucky’. They stored the cut inflorescences in cardboard boxes for 2, 4, 6 and 8 days under three different conditions: ‘in box at room temperature (23°C and 89% relative humidity)’; ‘in box under refrigerated storage (17°C and 94% relative humidity)’; and ‘control treatment’ i.e at room temperature condition (25°C and 77% relative humidity).They found that the optimum post-harvest attributes along with a shelf life of 7 days, were achieved under the first storage condition. However, the development of chilling symptoms (dark brown spots at the center and apex of bracts and at the conjunction of rachis and bracts) was noticed in the second storage condition, emphasizing the sensitivity of Heliconia to chilling. Paradoxically, Liju (2013) reported that a storage temperature of 17°C under packed conditions (details not found) is best for all Heliconia varieties, resulting in a shelf life of 8.6 to 11.6 days. Furthermore, brief storage of 4h at 20°C temperature before final storage also has positive effect on the post-harvest life of *Heliconia* (Liju, 2013). Darras (2020) also mentioned the significant problem of transporting *H. bihai* and other tropical ornamentals like *Alpinia, Dendrobium, Phalaenopsis, Strelitzia reginae*, and *Anthurium andraeanum* in mixed cargo with other low-temperature tolerant traditional cut flowers.

**Figure 1:**
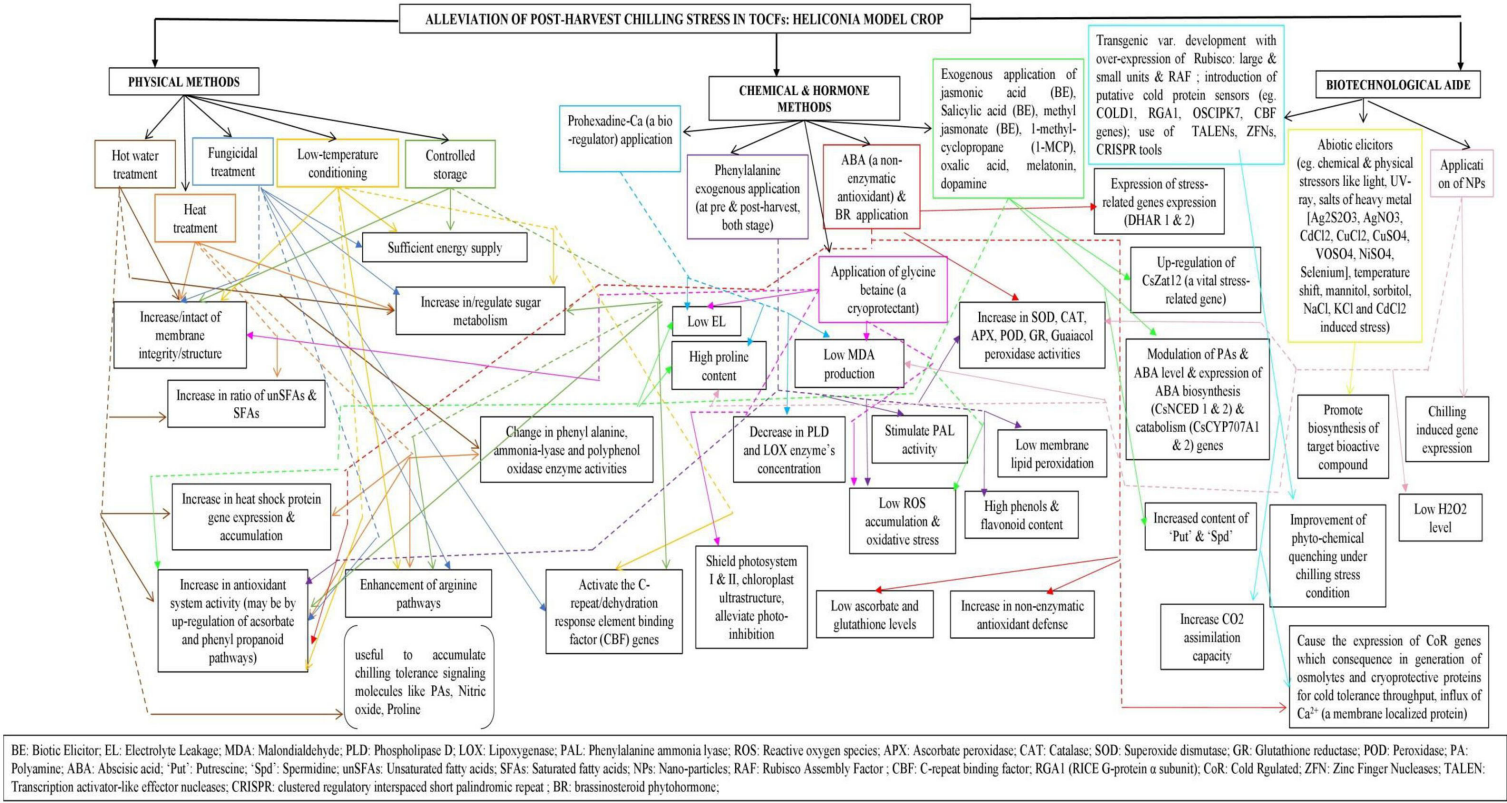
Schematic form of hypothetical strategies to obviate chilling injury (CI) in *Heliconia*. Arrows with black colored solid line indicate different treatments under principal methods. Multicolored treatment boxes have been used for segregation and easy identification purpose. Arrows with multicolored solid lines indicate the physiological impacts of individual treatments while the multicolored dotted lines only serve the purpose of connectors to reach different target distant square boxes. (Sources: Mirdehghan and Rahemi, 2004; Erkan et al., 2005; Nayyar et al., 2005; Kagale et al., 2007; Marton et al., 2010; Guo et al., 2012; Aghdam and Bodbodak, 2014; Gupta and Musunuru, 2014; Kim and Kim, 2014; Zhu et al., 2016; Zhao et al., 2017; Shi et al., 2018; Aghdam et al., 2019; Golding, 2019; Hahne et al., 2019; Halder et al., 2019; Vats et al., 2019; Zhang et al., 2019a; Ahn et al., 2020; Ahn et al., 2020; Darras, 2020; Ding et al., 2020; Liu et al., 2020; Salesse-Smith et al., 2020; Sogvar et al., 2020; Giovannini et al., 2021; Jiao et al., 2021; Song et al., 2022; Wei et al., 2022; Castro-Cegrí et al., 2023; Hui et al., 2023).

Very limited recent investigations and a few old investigations from several decades ago on Heliconia highlight the need for further research. In summary, storing of chilling-sensitive *Heliconia*at low temperature may disrupt various physiological processes such as water regulation, mineral nutrition, respiration, and overall metabolism. Therefore, novel techniques, should be developed to either breed chilling-resistant varieties or reduce their sensitivity to low temperatures in order to support the commercialization of Heliconia (**Figure 1**).

**Water Balance:** The water balance and post-harvest physiology of cut units are intimately related. The fundamental components of water balance include water uptake, loss, and accumulation, as well as the potential interactions between them (Sankat and Mujaffar, 1994). Water uptake plays a vital role in maintaining optimal hydration levels in cells (Costa et al., 2021), reducing abscission, senescence, and wilting (Seman and Rafdi, 2019; Costa et al., 2021), preserving the turgidity of floral units (Halevy and Mayak, 1981), and slowing down respiratory and metabolic activities (Kamemoto, 1962).

The water uptake capacity of *Heliconia* cut stems is relatively poor (Dolselman and Broschat, 1986; Criley and Paull, 1993). Therefore, it is crucial to carefully manage the water status in cut stems. According to Criley and Paull (1993), the water uptake rate of *H. psittacorum* var. ‘Lady Di’ and ‘Sassy’ decreases significantly after harvesting. However, the same researchers found that the average water uptake rate of the ‘Parakeet’ cut stem (without any foliage) was 3 mL after 15 days of harvesting. This result suggests that there may be a genotypic variation in the water uptake capacity of Heliconia. The findings of Carrera-Alvarado et al. (2021) also support these findings, as they observed differences in the water uptake potential of *H. wagneriana* (as low as 6%) and *H. psittacorum* x *H. spathocircinata* cv. ‘Tropics’ (0.08 - 0.11 mL g-1). It is characteristic of Heliconia stems to have low solution absorption (Jaroenkit and Paull, 2003; Carrera-Alvarado et al., 2020). The impaired solution uptake may be due to a close relationship between the diameter of the xylem vessels and susceptibility to embolism, where wider vessels are more prone to cavitations compared to narrow vessels (Arriaga-Frías et al., 2016).

Generally, in *Heliconia* cut flowers, the leaves are removed from the stem before commercialization because they tend to dehydrate quickly due to the high stomatal density. However, Ka-ipo et al. (1989) stated that there is a positive correlation between the number of attached foliage and water uptake in cut stems. This view is supported by several researchers (Broschat and Donselman, 1983b; Tjia and Sheehan, 1984; Criley and Broschat, 1992; Criley and Paull, 1993). They mentioned that removing all foliage and leaf sheaths from cut inflorescences significantly reduces water uptake rates, leading to a deterioration in fresh weight. The presence of leaves influences water uptake in plants, and the absorption rate is proportional to the number of leaves attached to the flower stem. However, in *Heliconia*, there are no vascular connections between the flower stalk and the leaves, unlike in other floral species. This contributes to the low water uptake after harvest (Carrera-Alvarado et al., 2020). Criley and Broschat (1992) also mentioned the physiological explanation that there are no upper ground vascular connections between the flower, peduncle, and leaves, which prevents water translocation and accumulation in the flower, thus not affecting its vase life. It is hypothesized that the immaturity of the basal intercalary meristem of the inflorescence peduncle at the cutting base may be the reason for this (Criley and Broschat, 1992). However, Ka-ipo et al. (1989) found that the intercalary meristem is located near the flower and not close to the basal end of the peduncle, except for the ‘Parakeet’ cultivar. Therefore, this hypothetical explanation is not suitable for this particular variety of *H. psittacorum*.

Whatsoever, water uptake may get hampered due to water quality, xylem occlusion by microorganisms, and deposition of pectin and phenols (Jedrzejuk et al., 2012). In general, the tap water is used as an easily available vase liquid medium and contains the common salts such as Ca(HCO_3_)_2_and CaSO_4,_ which determine water hardness. Van Meeteren et al. (1999) mentioned a threshold value of 60 mg/L for Ca, <25 mgL^-1^ for sulfate, and 30 mg/L for HCO3- in tap water. These researchers had raised an argument regarding the beneficial roles of distilled or deionized (DI) water as a control in post-harvest physiological studies. In their experiment on Chrysanthemum cut flowers, they found that DI water caused a sharp decrease in fresh weight of cut blooms after 1-3 days, while tap water did not show such an issue. Several researchers (Kirst and Bisson, 1979; Navarro et al., 2003) stated that DI water may mimic the normal physiological situation of an intact flower because xylem sap in intact plants contains various cations, anions, amino acids, and organic acids. However, Zimmermann (1978), in line with Van Meeteren’s argument, stated that drawing distilled water through stem segments progressively decreases the rate of conductance, but this can be eliminated by using tap water or a dilute osmoticum (e.g., 10 mM NaCl). Hutchinson et al. (2004) also confirmed the negative impacts of DI water on the post-harvest physiology of tuberose (Polianthes tuberosa) cut flowers. According to Halevy and Mayak (1981) and van Doorn (2012), this phenomenon may make water conductance in Heliconia stems more complex. Hence, this aspect should caution post-harvest researchers before using DI water for vase life improvement in Heliconia.

Different plant growth regulators (PGRs) such as BA and GA, as well as chemical substances like NaOCl, citric acid, sucrose, STS, HQC, and HQS, may also be used to maintain the components of water balance, as proven in *Lisianthus* (Musembi et al., 2013) and tuberose (Hutchinson et al., 2004) cut flowers. Tjia and Sheehan (1984) reported on the inefficiency of 8-HQC (antimicrobial agent) to improve the water uptake and prolong vase life in Heliconia. Microbial-induced stem plugging, often known as ‘physiological’ stem-plugging, occurs due to wounds or injuries at the stem end(Rogers, 1973). According to Rogers, the plugging may be caused by oxidative exudates resulting from the phosphorylation process of damaged cells, and the plugs may consist of pectin degradation products (Burdett, 1970). Furthermore, microorganisms can secrete enzymes (tannins, peroxidase) or other metabolic products that form viscous materials composed of calcium and magnesium salts of oxidized tannins. These materials move with the transpirational stream, causing pectin degradation and subsequent plugging (Rogers, 1973). This issue can be addressed by using an acidic preservative solution with a pH of 3-4.

Tjia and Sheehan (1984) found that the increased rate of water uptake during the nighttime is higher than during the daytime. This may be due to the high root hydrostatic pressure, which influences the flow of water uptake to the flower. For example, in *H. psittacorum*, a 2-fold increase has been observed. Folha et al. (2016) evaluated the benefits of periodic cutting (at intervals of 24 hours and 48 hours) of the base (1 cm in length) of peduncles of *H. psittacorum* cv. ‘Golden Torch’, as well as the renewal of vase water (deionized), in maintaining water balance components, fresh and dry mass, and post-harvest longevity. They found that these two strategies, at a 24-hour interval, optimize the restoration of water potential, leading to greater tissue hydration and maintenance of post-harvest quality. Similar results were also obtained in *H. wagneriana* by cutting the inflorescence peduncle every 2 days (Costa et al., 2015). After being removed from the plant, the flower starts to experience moisture stress. At this stage, the upward movement of the xylem water column draws air bubbles to accumulate at the cut end. These air bubbles, lodged against the cross wall of one of the xylem vessels, impede moisture flow and consequently reduce water uptake. Vacuum infiltration of the stem end, the use of acidified water (pH 3.5), calcium nitrate, and enzyme inhibitors (such as azide and DNP) promote normal water uptake and help regain turgidity (Rogers, 1973). Except for Anthurium, no significant investigations regarding the linkage between water balance and vase life improvement have been reported for other tropical ornamental plants. However, relevant reports are summarized in **Table 5** to maintain the breadth and conciseness of the article.

## 3 Preservative solutions

The detachment of cut blooms, the actively metabolizing plant part, from the mother plant deprives them of the raw materials that are essential for a myriad of metabolic processes. Therefore, the need arises to externally supply these naturally available essentials. Generally, water, anti-senescent substances (e.g., benzimidazole, kinetin, benzyladenine, etc.), and respirable substrates (i.e., sucrose, glucose) are highly demanded substances required to maintain excised blooms in a decorative form (Rogers, 1973). Additionally, various chemicals (e.g., STS [silver thiosulfate], AgNO3 [silver nitrate], citric acid, 8-HQ [hydroxyquinoline], boric acid, Al2(SO4)3 [aluminum sulfate], sodium hypochlorite, NO [nitric oxide]-based donor compounds, 1-MCP [1-methyl cyclopropane], etc.) and growth regulators (e.g., gibberellins, cytokinins, auxins, etc.) can be provided through pulsing (momentary treatment), holding (vase) solutions, or postharvest fumigation treatments (Badiyan et al., 2004; Janowska and Andrzejak, 2023) with the aim of improving the post-harvest life of cut blooms. In the following sections, the functions of the aforementioned substances to prolong the post-harvest life of Heliconia blooms are delineated.

### 3.1 Chemicals based preservatives

Based on the bibliographic searches, the overall idea of a low water uptake capacity by the *Heliconia* inflorescence’s peduncle has been acknowledged. It has been found that this problem cannot be improved by the use of various vase life-extending chemical components, either alone or in combination. However, exceptional cases have also been discovered. The research outcomes of Broschat and Donselman (1983a); Ka-ipo et al. (1989), and Tjia and Sheehan (1984) can support the aforementioned statement.

8-hydroxyquinoline citrate or sulfate (8-HQC or S), sucrose, silver thiosulfate (STS), dithiothreitol (DTE), citric acid, etc., are commonly used preservatives in Heliconia (Broschat and Donselman, 1983b; Bredmose, 1986; Ka-ipo et al., 1989; Whittaker, 1993; Malakar et al., 2019). Among these, 8-HQ salts and sucrose possess multifarious potential properties, such as anti-desiccant and anti-microbial components, and the ability to maintain balanced water content. The preservative-based solutions prepared using non-deionized water containing less than 200 ppm of total dissolved salts usually result in satisfactory water uptake, as reported by Rogers (1973). Costa et al. (2015) found improved post-harvest longevity in *H. wagneriana* after pre-treatment with 10% and 20% sucrose solutions and holding solutions of 30 and 75 mg/L of AgNO3. Liju (2013) discovered that pulsing with a solution of 5% sucrose + 200 ppm 8-HQ for 6 hours, followed by a holding solution of 5% sucrose + 100 ppm 8-HQ, extended the post-harvest period in seven varieties of Heliconia. Sarkar et al. (2022) also obtained similar results for different Heliconia genotypes using 8-HQC-based holding solutions. Recently, Malakar et al. (2019) evaluated the consequences of different vase solutions on the post-harvest durability and quality of Heliconia cut inflorescences available in West Bengal, India. They used silver nitrate (AgNO3): 1500 ppm, calcium chloride (CaCl2): 750 mg/L, citric acid: 200 mg/L in combination with 8-HQC: 500 mg/L and sucrose: 2%. The treatment combinations of AgNO3, CaCl2, and citric acid, along with 8-HQC and sucrose, were found to be the most effective for all the Heliconia genotypes, prolonging vase life (up to 7 days on average, compared to the control of 7-8 days) and improving other features such as pigment retention, solution uptake rate, and enzymatic activities (high catalase [CAT], peroxidase [POD], and low lipid peroxidation). However, they mentioned that the performance may vary depending on the species and variety. Additionally, the spray of bovine serum albumin (BSA): 50 mg/L was found to prolong vase life by almost 2-fold in ‘Golden Torch’, as reported by Mangave et al. (2013).

Holley (1960) estimated that as much as one-third of a flower’s shelf life may be influenced by its pre-harvest environment, while the remaining two-thirds are determined by postharvest maintenance. One of the reasons for the qualitative deterioration of cut blooms is the depletion of respirable substrates, although there is a connection between the amount of reserved dry matter content and the shortage of respirable substrates (Rogers, 1973). According to the same researcher, exposing the excised units to incidental light increases the photosynthetic capacity and production of photosynthates, indirectly alleviating the scarcity of respirable substrates. A carbon dioxide-enriched growing environment (pre-harvest) also influences the storage of more photosynthates (Shaw and Rogers, 1964; Mattson and Widmer, 1971).The physiological process known as “respiratory metabolism” is closely associated with the post-harvest improvement of cut blooms. A shift in respiratory quotient is observed in isolated blooms, with Hew and Yip (1987) estimating the highest respiratory quotient of 1.0 (in excised petals) compared to 0.5 (in *in situ* blooms at the tight bud stage) in Aranda orchid petal cells. They also mentioned that in cut blooms, carbohydrate metabolism predominantly occurs via the EMP pathway (the synthetic conversion of glucose to pyruvate), and there is a shift towards cyanide-sensitive respiration. Additionally, sucrose not only serves as a source of carbohydrates (Malakar et al., 2019) but also helps protect the ultrastructure of chromoplasts, resulting in pigment retention (Singh et al., 2008). Sucrose also aids in maintaining water balance, keeping the bract cells turgid by influencing osmotic pressure (Halevy and Mayak, 1981).While Tjia and Sheehan (1984) mentioned that 8-HQC or 8-HQS does not have any impact on solution uptake or the elimination of microbial occlusion, Malakar et al. (2019) found the beneficial impact of this germicide on extending vase life.

According to Subhashini et al. (2011), Ag++ ions help to prolong the vase life, while Ca++ ions reduce the respiratory rate and contribute to cell wall toughness, preventing cell breakage or collapse. The broad antimicrobial effects of AgNO3 are well-known, as Ag++ ions replace the hydrogen cations (H+) of sulfhydryl or thiol groups (-SH) on the cell membranes of bacteria, resulting in membrane integrity loss and cell death (Feng et al., 2000; Li et al., 2020; Elatafi and Fang, 2022). Several researchers (Jiang et al., 2004; Foldbjerg et al., 2009; Elatafi and Fang, 2022) have revealed that Ag nanoparticles (Ag-NPs) are more effective than other forms of Ag because they possess a larger surface area-to-volume ratio, making them more efficient as a biocide. Additionally, Ag-NPs have lower toxicity effects and optimize the content of soluble solids (SSC), titratable acidity (TA), malondialdehyde (MDA), as well as the activities of polyphenol oxidase (PPO), pyrogallol peroxidase (POD), and pectin methylesterase (PME).

The pH of the solution also has a significant impact on vase life. A neutral or alkaline pH is not suitable for maintaining satisfactory post-harvest quality (Reid and Kofranek, 1980). The pH of plant cell sap is generally between 3-3.5 (Khan et al., 2009; Gupta and Dubey, 2018); therefore, an acidic solution or the addition of organic acids like citric acid can yield good results. Low-pH water (pH 3.5) travels faster in the water-conducting system (xylem), thereby preventing or reducing wilting during the post-harvest stage. Commercial rehydration solutions, such as Hydraflor, often contain sufficient citric acid to lower the pH of the vase solution to 3.5 (source: https://ag.umass.edu/greenhouse-floriculture/fact-sheets/sugar-acidity-in-preservative-solutions-for-field-grown-cut).

Some eco-friendly solutions could also serve the purpose of extending the vase life, as evidenced in the case of Heliconia ‘Golden Torch’. Shokalu et al. (2021) studied the impact of aloe vera (*Aloe barbadensis* Miller.) and moringa (*Moringa oleifera* Lam.) solutions on prolonging the display life of the said Heliconia variety. They found that the combination of aloe vera solution (5%) along with 4% sucrose could improve the water balance components, resulting in a 67.4% increase in open bracts and 78.9% relative water content (RWC). In the case of Anthurium, the use of *Stevia rebaudiana* extract at a concentration of 0.1 mgL^-1^along with 10 mgL^-1^ of nano-silver had been found to enhance the water uptake rate after fifteen days of placing the cut bloom in the solution (Amin, 2017). The scientific and commercial approaches towards using organic extracts as potent preservatives for cut tropical ornamentals are still in the early stages.

Regarding other tropical ornamentals, except for *Strelitzia* and *Anthurium* (**Table 5**), very limited published reports have been observed. For example, in the case of *Globba*, several researchers (Criley, 2014; Branney, 2005; Chuengpanya et al. 2016) have mentioned that the longevity of its bracts may last up to 1-1.5 months, but scientific authentication is lacking. Despite having significant cut flower attributes, there are no scientific reports available, although an article by Aung et al. (year missing) on “post-harvest quality and vase life of ornamental cut flower *G. orixensis*Roxb.” has been found, but its accessibility is limited. The status of Hedychium is also the same; the frequent emergence of new flowers and its limited use as a cut flower may have hindered post-harvest researchers from conducting investigations on standardizing chemical formulations for bud opening and extending vase life. The current scenario emphasizes the importance of conducting research on Hedychium to bring it to the forefront and establish its use as a commercial tropical cut bloom.

### 3.2 Growth regulators based preservatives

Mangave et al. (2013) investigated the role of growth regulators spray (GA, BA, and Alar [synonymously Daminozide]) in extending the quality and post-harvest life of the ‘Golden Torch’ cultivar of *H. psittacorum x H. spathocircinata*. The spray of GA: 100 mg/L had yielded satisfactory effects in this case by decreasing enzymatic activity and lipid peroxidation, while also improving factors such as the percent absolute integrity (PAI) of the bract cell membrane (which delays bract cell death), enhancement in inflorescence fresh weight, vase life (2-fold increase over control), and removal of oxidative stress. Malakar et al. (2019) also found the positive impact of GA3 at a dose rate of 80 ppm on vase life extension of different genotypes of Heliconia.

BA is a synthetic cytokinin (CK) (Paull and Chantrachit, 2001; e-source: https://www.acs.org/molecule-of-the-week/archive/b/6-benzyladenine.html) that has been reported to increase the vase life of diverse tropical ornamentals, including Heliconia. Paull and Chantrachit (2001) stated that the use of BA, in both spray and dipping forms, could extend the vase life of *H. psittacorum* var. ‘Sexy Pink’ inflorescences up to 21 and 18 days, respectively. Similarly, the use of BA in these forms could retain the keeping quality of ornamentally significant leaves and flowers of the ‘Andromeda’ variety for up to 32 and 31 days, respectively. BA helps delay both bract darkening and abscission. Another study on the effect of BA was carried out by de Moraes et al. (2005) in *H. latispatha*. They found a linear increase in vase life with increasing concentrations of BA spray (100, 200, and 300 mg/L). The longest vase life, 1.85-fold increase, was obtained at a concentration of 300 mg/L of BA. The dipping or spray of BA at a dose rate of 200 mg/L also extended the vase life of the ‘Nickeriensis’ variety of Heliconia by about 8 days, as reported by Whittaker (1993). According to Whittaker (1993) and Costa et al. (2021), the treatment using BA at a concentration of 200 mg/L increased the vase life of Heliconia genotypes (var. ‘Sexy Pink’ and ‘Andromeda’) by 1.2 to 2.5-fold.

GA reduces senescence rates by regulating cell membrane permeability and protein degradation (Shaul et al., 1996) and by influencing the action of ABA (Kumar et al., 2014; Costa et al., 2016). Cytokinins (CKs) and GAs are considered inhibitors of aging; however, unfortunately, their content in plant tissues decreases during the aging process, while the levels of regulators that accelerate aging, such as ethylene, salicylic acid (SA), brassinosteroids (BR), abscisic acid (ABA), and jasmonic acid (JA), increase (Asami and Nakagawa, 2018; Janowska and Andrzejak, 2022). During the aging of petal or perianth cells, active membrane-damaging enzymes, proteolysis, accelerated breakdown of pigments, and a large amount of free radicals contribute to the destruction of cell components (Rogers, 1973). A high content of reactive oxygen species (ROS) causes oxidative stress, leading to damage to cellular macromolecules and membranes, as well as increased lipid peroxidation (Janowska and Andrzejak, 2022). Application of BA and GA3 significantly reduces protein degradation (Hayden, 2003), and both act as pigment protectors (Rabiza-Świder et al., 2012). Moreover, pre-soaking plant parts in GA3 solution before planting and harvesting stages noticeably enhances the content of hydrocarbons, especially fructose and glucose, thereby improving post-harvest life (Janowska et al., 2022; Janowska and Andrzejak, 2022). In other related tropical ornamentals, the use of growth regulators has been found to be limited (**Table 5**).

## 4 Other strategies to improve after-harvest life

Since water balance is the ultimate factor in preventing desiccation injury and subsequent senescence, maintaining the rate of water uptake and minimizing evaporative water loss are two vital factors (Criley and Broschat, 1992). Given the circumstances, the use of anti-transpirants can be a viable solution.

Ka-ipo et al. (1989) mentioned that dipping ‘Parakeet’ types of *H. psittacorum* in a ‘Wilt Pruf’ solution (dilution rate 1:10) or ‘Wax’ solution (dilution rate 1:4) could increase the vase life by up to 36%. Dipping ‘Nickeriensis’ type in a ‘Folicote’ solution (dilution rate 1:40) could also extend the vase life by around 26% as reported by Whittaker (1993). Carrera-Alvarado et al. (2021) conducted a study on the impact of waxing and salicylic acid (1mM) treatment on the post-harvest life of H. wagneriana under low temperature (13°C and 84% RH) conditions. They found that the bract tissue of waxed peduncles showed optimal enzymatic activity, low oxidative stress, good water accumulation (maintenance of bract turgidity), and a satisfactory shelf life of an extra 2-3 days compared to 9 days under control conditions. In the case of the ‘Golden Torch’ cultivar, graded concentrations of wax emulsions (0.25%, 0.50%, 0.75%, and 1%) were found to enhance the shelf life from 10-11.78 days, while the fresh weight increased by 48% (Powar et al., 2014). However, several researchers [Criley and Broschat, 1992, Broschat and Donselman (1987)] expressed concerns about the limited efficacy of waxing in extending the vase life of Heliconia genotypes. This may be due to the inability to cover the entire bract surface, especially the groove areas, while the waxy surface of the bract cuticle could be another reason, as reported by Criley and Paull (1993).

Another effective strategy may be hot water treatment, as the deterioration in keeping quality can also be caused by disease infestation. Therefore, this treatment may minimize the problem. In the case of *H. chartacea* var. ‘Sexy Pink’, *H. caribaea* (Red), and *H. psittacorum* (Red), hot water treatment (49°C temperature for 12 minutes) has been found to increase the keeping quality for a few days (Criley and Broschat, 1992). However, no further details regarding the threshold level of temperature and duration have been reported by any researchers for Heliconia.

Among other tropical ornamentals, the application of waxing treatment has been noticed in Strelitzia and Etlingera. For example, de Paula et al. (2021) evaluated the impact of canauba wax coating (20% and 40%) on the foliage of *S. juncea* and *S. reginae*, resulting in an extended post-harvest longevity of 16th day for *S. reginae* and 18th day for *S. juncea*, low leaf mass loss percentage, and minimal visual quality impairment. Additionally, the application of 3% carnauba wax on the bracts of *E. elatior* var. Porcelana (at semi-open and fully open stages) effectively maintained water balance, carbohydrate content (starch and total soluble sugar), promoted bract expansion, and flower opening (Mattos et al., 2017; Mattos et al., 2018).

In Strelitzia, the major post-harvest problem is the development of saprophytic mold, leading to floret desiccation and bract darkening (Jaroenkit and Paull, 2003; Balas et al., 2006; Koley, 2013; Criley, 2014). The secretion of mucilage during anthesis encourages Botrytis growth, while post-harvest nectar and slime production facilitate saprophytic mold growth (Jaroenkit and Paull, 2003; Criley, 2014). Jaroenkit and Paull (2003) suggested dipping Strelitzia inflorescences in a solution of benomyl or thiobendazole (200 mg/L concentration) to address this issue.

‘Geotropic curvature’ has been mentioned as a serious concern in *Alpinia* (Criley and Paull, 1993). Dipping red ginger inflorescences in TIBA (tri-iodobenzoic acid; an auxin movement inhibitor) could be useful in controlling the geotropic curvature, as reported by Chantrachit (1999). Hot water treatment (49°C for 12 minutes) and maintaining a vertical posture during shipment may be other viable remedies (Hara et al., 1997). In the case of Heliconia, no such post-harvest issues have been documented.

To restrict insect and pest infestations, irradiation treatment of 250 Gy may be useful (Sangwanangkul et al., 2008). To minimize minimal irradiation injury, hot water treatment (40.0-47.5°C) for a duration of 20-30 minutes may be applied, although the physiological basis for improvements after heat treatment is yet to be unraveled.

Topolines (Ts), ionic liquids, and quaternary ammonium salts with selected organic cations and GA3 anions have been used in florist greens (ornamental foliage) to improve post-harvest longevity (Janowska and Andrzejak, 2022). Therefore, their implementation may also be introduced in Heliconia.

The Controlled Atmospheric Storage (CA) system, which relies on low levels of O2 (0.5-1% for flowers, but not 0, as it may cause undesirable anaerobic breakdown reactions) and increased levels of CO2, can be implemented to reduce respiration rates and preserve respirable substrates during post-harvest storage (Rogers, 1973). This approach may also be applied to Heliconia. All tropical ornamentals, including Heliconia, are ethylene insensitive, but an exception has been found in Strelitzia. According to Bayogan et al. (2008), Strelitzia is not highly sensitive to ethylene, but its longevity may be affected by exogenous ethylene exposure. Pre-treatment using 500 ml L-1 of 1-MCP and 0.2 mM STS for 6 hours at a temperature of 20°C can reduce ethylene-related injuries, as reported by Macnish et al. (2009), who exposed Strelitzia inflorescences to 1 µL L-1 of ethylene for 24 hours. This may be useful in preventing any undesirable physiological changes that may occur in tropical ornamentals.

### 4.1 Post-harvest handling

At this phase, the harvested produce is subjected to cleaning and grading processes, which are considered preceding steps to final packaging. Cleaning is a crucial step in post-harvest handling, particularly during export. In Heliconia, insect disinfection and additional cleaning and treatment steps are typically followed because the floral structures (bracts) of Heliconia provide natural hiding sites for many insects (Jaroenkit and Paull, 2003). In Hawaii, the conventional hand wash along with insecticidal soap is usually employed, and washing in a detergent solution followed by rinsing with pressurized water flow can be useful not only for cleaning purposes but also to remove field heat. For *Heliconia* inflorescences’ disinfection, a solution of 50% Diazinon 40 WP (160g/100 L-1 of water) + 0.18 L of light volck oil or a solution of 57 EC malathion (1 ml L-1) can be used (Nowak and Rudnicki, 1990; Criley and Paull, 1993). Hot water treatment has also been found to be effective for disinfection purposes (Hara et al., 1997). However, recent advanced research approaches in tropical ornamentals, such as the usage of ultraviolet-C radiation for sanitation purposes, are not yet available, although they are being used to characterize the effects on the sanitary quality of castor oil seeds (de Araujo et al., 2019).

Regarding *Strelitzia*, cleaning before the packaging of the inflorescence is another essential step. The structural characteristics of *Strelitzia* inflorescence, which are inappropriate as habitats or resting places for various insects and pests, have minimized the need for mandatory cleaning, except for simple washing with water to remove field dust and heat (Jaroenkit and Paull, 2003).

In terms of grading standards for *Heliconia*, to meet the “Hawaii Fancy Grade” standards, each inflorescence must have at least 2 open bracts, while other units must be well-formed, and the minimum length of the inflorescence peduncle must be 15 cm (Hawaii Department of Agriculture, 1972). For *Anthurium*, grading is related to descriptive and visual aspects of the spathe and spadix, including color, shine, turgidity, and the occurrence of spots and necrosis (Cuquel and Polack, 2010). The standard grading of *Anthurium* cut blooms based on spathe width is as follows: >15 cm - Grade (G1), 13-15 cm - G2, 11-13 cm - G3, 9-11 cm - G4, 7-9 cm - G5, 5-7 cm - G6, and >5 cm - G7 (Ng et al., year missing). Several scientific reports on digital vision-based grading systems for *Anthurium* have been observed (Hemming et al., 2010; Soleimanipour et al., 2019; Soleimanipour and Chegini, 2020), while this aspect is completely unknown for other tropical ornamentals. In summary, there is a lack of investigation reports on the characterization of mandatory export grading systems for tropical ornamental cut flowers, highlighting the importance of conducting intensive research on this topic in the near future.

Overall, proper post-harvest handling can affirm the retention of all essential aesthetic attributes which along with increase in market importance could also broaden the participation potential of group of tropical flowers’ in value-added based enterprises like in bio-color and alternative dietary sources [i.e edible ornamentals; eg. edible status of bracts of ‘Torch Ginger’ (Lekawatana and Pituck, 1998)] sectors, in ‘green chemistry’ industries [eg. use of *Alpinia* plant part’s extract for ‘green synthesis’ of nano-particles (Zhang et al., 2019b; Shinde et al., 2021)] etc. But the negligence has impeded the involvement of this specialty cut flowers’ group in value-added studies as mentioned by Kreissig (2019) and Quinaya and d’Almeida (2019).

## 5 Advancement in packaging: potent shield of post-harvest quality

**Figure 2:**
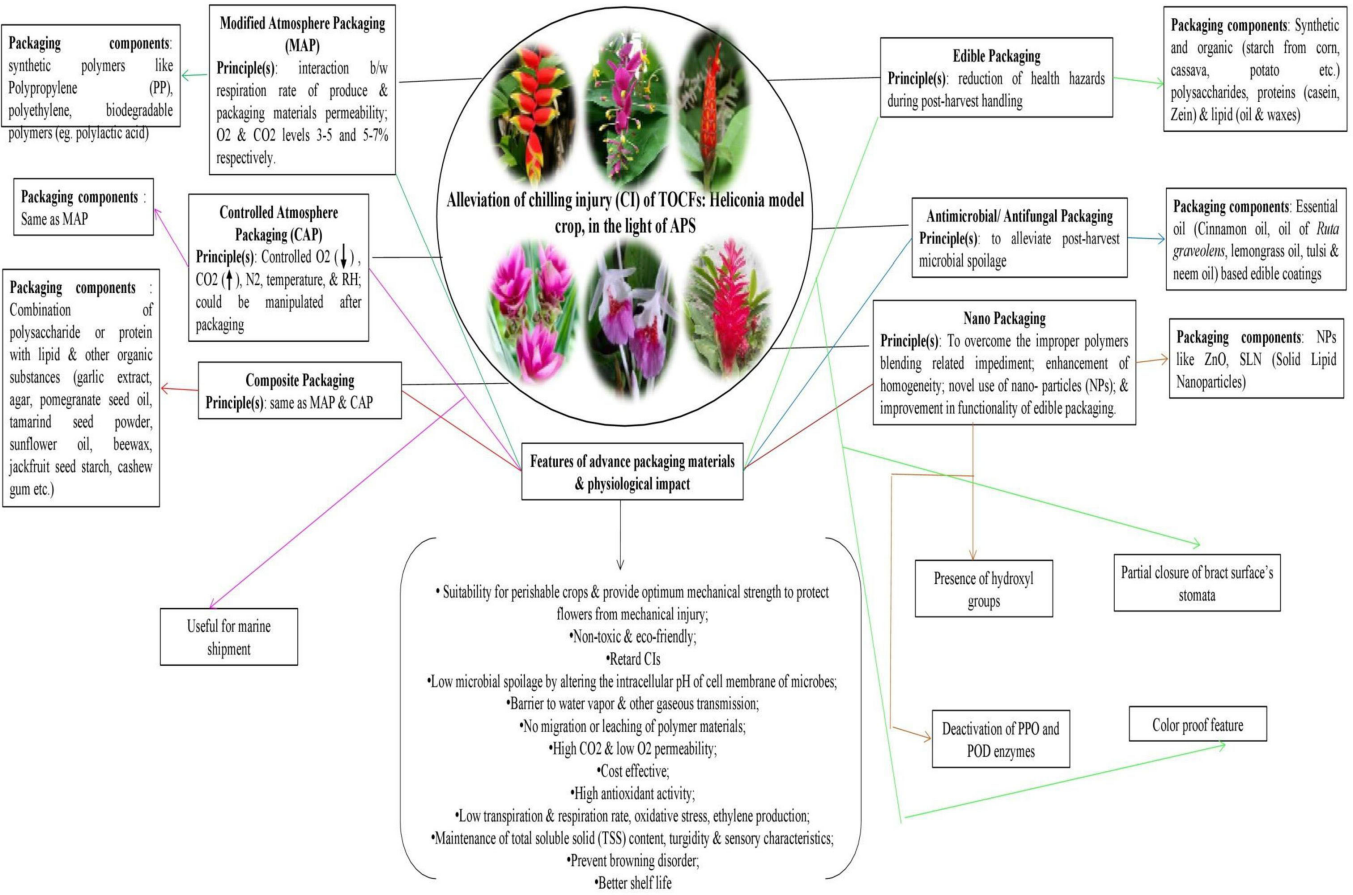
Graphical abstract of hypothetical strategies to alleviate CI in Heliconia resorting advance packaging system (APS) Source: Etemadipoor et al., 2020; Yadav et al., 2022. The ‘Solid’ lines are merely the connecting lines. Black solid lines indicate the key advanced packaging systems. Other multi-colored solid lines show the packaging components of the respective packaging systems. Other multi-colored arrows show the other details of the respective packaging systems.

**Figure 3:**
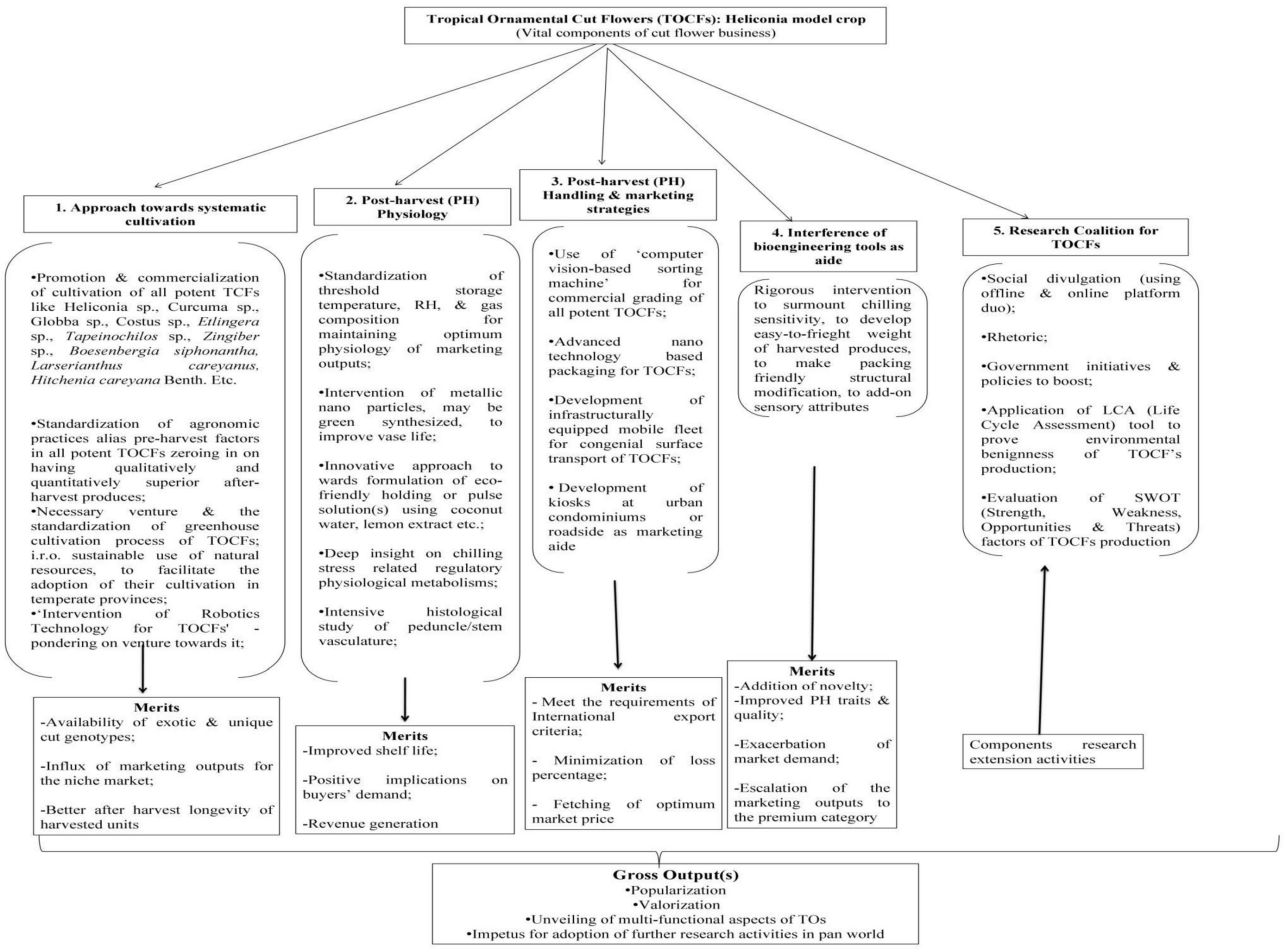
Future thrust areas on the Tropical Ornamental Cut Flowers (TOCFs) and its subsequent expected outcomes.

**Figure 4:**
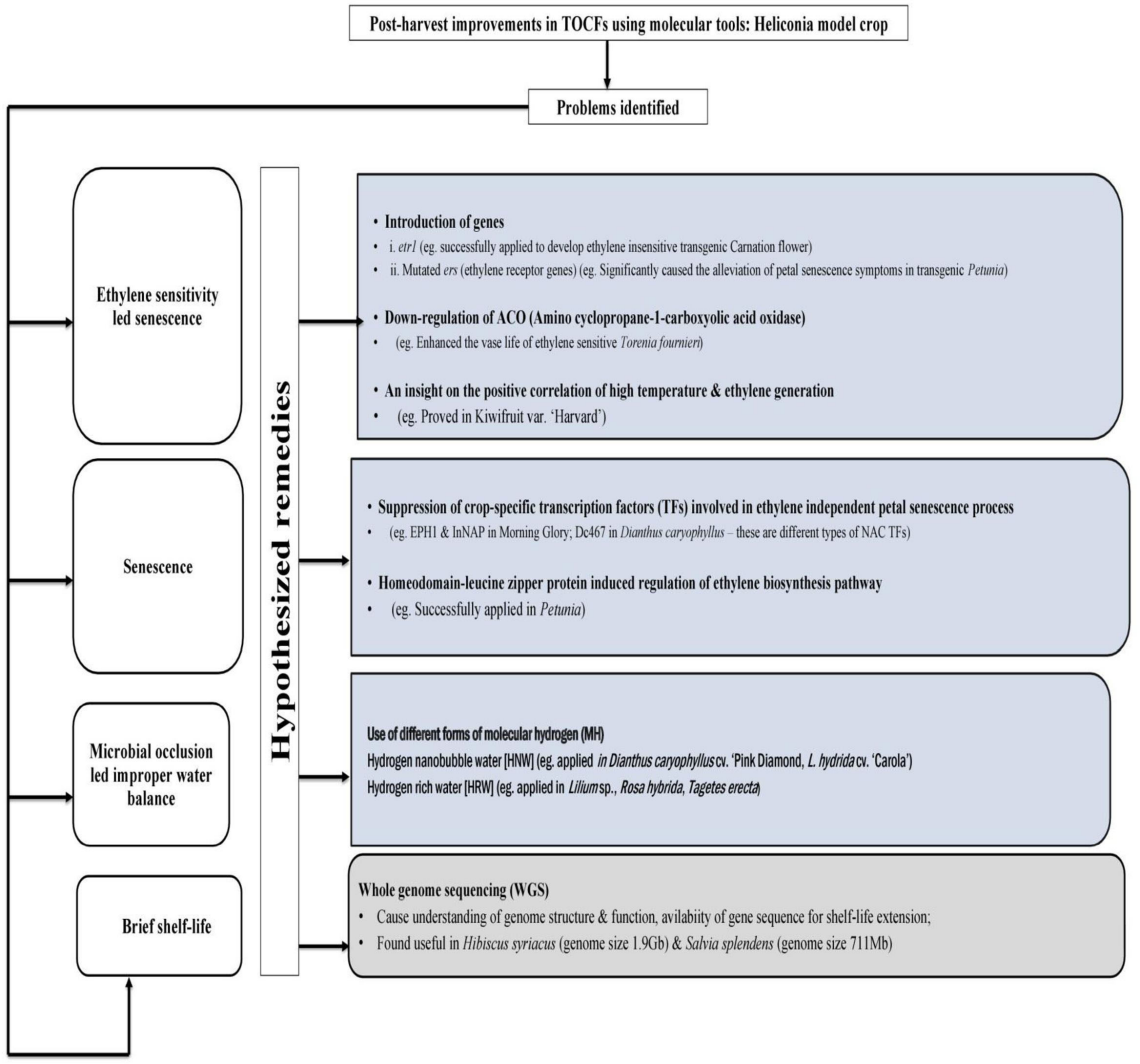
Hypothesized strategies for post-harvest improvement by biotechnological interventions in Heliconia. Sources: Bleecker and Schaller, 1996; Aida et al., 1998; Antunes and Sfakiotakis, 2000; Kosugi et al., 2002; Shibuya et al., 2014; Yin et al., 2015; Kim et al., 2017; Ren et al., 2017; Zhu and Liao, 2017; Deng et al., 2018; Woo et al., 2018; Yagi, 2018; Ahn et al., 2020; Carrera-Alvarado et al., 2021; Costa et al., 2021; Giovannini et al., 2021; Verma et al., 2021; Fang et al., 2021; Nguyen and Lim, 2022.

Packaging acts as a protective cover to absorb shock during the transport of cut flowers. Not only that, it helps maintain the optimum physiological condition of the flowers throughout the transportation distance, ensuring their quality remains intact. Appropriate packaging, combined with pulsing, is helpful in ensuring fresh quality for consumers and extending the vase-life of the flowers (Senapati et al., 2016). However, the unusual structure, extravagant size, and weight of tropical ornamentals, including *Heliconia*, pose significant challenges in terms of packaging and transport. Viable measures to meet this challenge are almost absent, with only a handful of reports available (**Table 6**). Due to the large size and heavy weight of Heliconia inflorescences, large containers packed with moistened shredded papers are used to maintain humidity and prevent bruising (Criley and Paull, 1993). Smaller *Heliconia* species like *H. psittacorum* and *H. angusta* are usually packed in bunches of 5 and 10, respectively, while larger and heavier species are packed individually in cartons, with plastic film or net sleeves used to minimize bruising (Criley and Paull, 1993). Liju (2013) reported that packing Heliconia inflorescences with a wet cotton plug at the peduncle end and using polythene lining yields the best results. For Strelitzia, cardboard containers sized 102x43x28 cm are generally used for packaging cut units (Criley and Paull, 1993). To keep pace with the recent demand for tropical cut flowers, the adoption of advanced packaging systems such as Modified Atmosphere Packaging (MAP), Controlled Atmosphere Packaging (CAP), Composite Packaging, Antimicrobial/Antifungal Packaging (AP), Edible Packaging (EP), and Nano Packaging (NP) is crucial (Yadav et al., 2022). However, none of these packaging systems are currently in practice for tropical cut flowers, although they are commonly used in fruit crops. These advanced packaging systems rely on the principles of reducing the rate of oxygen consumption, maintaining high CO2 concentration to prevent ethylene production, and extending the shelf life of the produce (Murmu and Mishra, 2018a and Mangaraj et al., 2014; Murmu and Mishra, 2018b; Etemadipoor et al., 2019; Wang et al., 2020). They also maintain the proper gaseous environment around the packed produce, reduce respiration and biochemical reaction rates, and impede transpiration through the use of packaging materials such as polymeric films like PP, LDPE, etc. (Forato et al., 2015; Mamede et al., 2016; González-Reza et al., 2018; Teixeira, 2020), thus keeping the packed produce fresh and turgid (Yadav et al., 2022). Due to stringent environmental legislations, the use of plastic-based films has been minimized, and the usage of biodegradable films or edible films or coatings (commonly used in fruits like guava) is being encouraged (Yadav et al., 2022). The implementation of these techniques for Heliconia and other related species appears to be absent, possibly due to a lack of knowledge, negligence, and suppression, in addition to other physiological bottlenecks mentioned in preceding sections. Since chilling stress is one of the major setbacks in the export of tropical cut flowers, the use of novel or advanced packaging systems resistant to chilling stress should be explored for their export worldwide (**Figure 2**). Furthermore, the weight of tropical flowers, especially *Heliconia*, significantly restricts their export due to increased air freight charges. To maintain an optimum cost-benefit ratio, air transport is found to be nonviable, and the adoption of surface or marine transport is unquestionable due to the prolonged duration. To overcome these hindrances, weight reduction methods, the development of low-weight *Heliconia* varieties, or the use of advanced and equipped means of transport need to be devised. In this context, the use of a novel dried form of cut units, known as ‘Dehydrated cut inflorescences,’ may be a viable way to reduce the weight of *Heliconia* cut flowers, although this aspect remains unexplored. Additionally, the employment of cutting-edge molecular aids and functional technological advancements may facilitate the export of *Heliconia* seamlessly. Summarily, significant improvement in packaging of tropical flowers ought to be one of the imperative aspects of several future thrust areas (**Figure 3**) while active utilization of frontier bio-technological aides may also be a viable option to achieve the target in totality (**Figure 4**).

**Table 6:** State-of-art of packaging and transport in tropical cut flowers.

Name of flowers	Packaging methods adopted	Packaging materials	Details of packaging	Impacts of packaging	Mode of transport& other details	Remarks	Source(s)
*Alpiniapurpurata*	MAP	*PP plastic; large Containers*	Packed with moistened shredded newspaper to prevent bruising and to maintain high humidity within the carton	*Longer freshness period (7.77 days), lowest weight loss (0.71%), optimum freshness (sensory score of 4), capacity of holding solution uptake increased (4.81mL/stalk/day)*	Air freight	The text is illegible	Marsetyowati, 2014; Akamine, 1976; Kobayashi et al., 2007
*Anthurium*	Cartoon packaging (size 21.6 x 50.8 x 91.4 cm; 27.9 x 43.2 x 101.6 cm)	Corrugated cardboard	The basal stem end of cut units are inserted in water filled rubber balloon to render optimum hydration; Wax paper sleeving is used	Prolonged freshness	Surface transportation;	considered as hard ornamentals than tropicals, especially tropical orchids like Vanda; hence, surface transport had been preferred over air transport from Hawaii to US	Akamine, 1976
*Strelitzia*	*Containers packing*	*-*	packed with moistened shredded newspaper to prevent bruising and to maintain high humidity within the carton	Prolonged freshness	Surface transportation& Air freight both	Considered as hard ornamentals than tropicals, especially tropical orchids like Vanda; hence, surface transport had been preferred over air transport from Hawaii to US	Akamine, 1976
*Vanda*	MAP	PP plastic; sometimes corrugated cardboard cartoons	reduced pressures;gases used N2 or CO2;atmospheric pressures 125-190 mm Hg;O2 concentration ranges from 3.45%-5.25%, 3% O2 in air may be modified with N2 or 3% CO2 in air	Long shelf life	Air freight; shipped as intact flowers or as leis;	Ethylene producer; packaging materials must be gas impervious and sufficiently durable to withstand any “ballooning” effect due to decrease in atmospheric pressures in flight.	Akamine, 1976
*Cattleya*	Cartoonpackaging of variable sizes	Corrugated cardboard, Foam box & Plastic vials	–	Long shelf life	Air freight	–	Akamine, 1976; De, 2020
*Cymbidium*	Carton packaging of variable sizes	Corrugated cardboard, Polypropylene-150 gauge;Cellophane; LDP-100 gauge; HDP -150 gauge; Newspaper	–	Long vase life	Air freight	–	Akamine, 1976; De, 2020
*Dendrobium*	Carton packaging of variable sizes	Corrugated cardboard, Foam box & Plastic vials, Cellophane	–	Long shelf life	Air freight		Akamine, 1976, De, 2020
*Etlingeraelatior* (Torch zinger)	–	–	–	–	–	packaging & transportation are very limited for high mass of inflorescences (over 1 kg)	Gonçalves et al., 2014; Araújo et al., 2018; Loges et al., 2008
*Zingiberspectabile* (Beehive zinger)	Carton packaging of variable sizes	Corrugated cardboard	Individual cut units are wrapped by plastic bag or mesh; lining by polyethylene film in b/w the cut flowers are made; bactericidal treatment at pre-packaging phase	Better shelf life	Refrigerated (15-18^0^C & 90% RH) surface transport	–	da Silva Veira et al.,2014
*Strelitziareginae*	102x43x28cm sized cardboard containers	Corrugated cardboard	Usually packed in bunch of 5; newspaper used as wrapper material; pre-packing and transport dipping treatment in benomyl: 200mgL^-1^ solution to curb nectar and slime production & saprophytic mould growth	Long shelf-life	Air freight	–	Criley and Paull, 1993; Jaroenkit and Paull, 2003
*Curcuma* cv. ‘Chiang Mai Pink’	MAP, carton packaging	PP film; fiberboard cartons	Coating of cut stem end by mixture of 25 ppm BA & GA3 each before packaging	Long shelf life	No information given	–	Yimphak and Chanasut, 2009
*Globbabulbifera*Roxb.	Carton packaging	wooden box, newspaper, the leaves banana	–	Better longevity	Surface transportation in Malaysia	–	Win, 2020; Criley, 2014

-, No information.

## 6 Conclusion

Tropical cut flowers are vital components of the floriculture industry. However, their commercialization has been hindered by non-commercialization, unprofessionalism, and various loopholes in research and extension activities, as well as in physiological studies related to these flowers even after possessing unique appearance and diverse range of colors. Moreover, the unconventional structure of tropical cut flowers, along with environmental filters and physiological intricacies, has also limited their widespread cultivation.

These have caused the impetus to pen up all available published scientific information(s) as well as knowledge-based views explicitly on Heliconia which is a chosen model crop as well as the ambassador of TCFs group. This review summarizes all possible pre-harvest, harvest and post-harvest factors which are the potent determiner of after-harvest life of them. The major issues regarding tropical flowers are the ‘low temperature sensitivity’ which restricts to ferry with other traditional cut flowers and ‘packaging’ for the out of the turn shape, size and weight. The hypothetical measures to rid both the said concerns have also been highlighted which may hint some useful cues for the conductance of further investigations. Furthermore, the cursory overview on contextual aspects of other related tropical ornamental members may also assist the readers to associate with the current realistic research scenario. Summarily, our approach may be considered as an initiative for the promotion of this specialty group of flowers.

Successful mitigation of all concerns and the realistic application of all associated endeavors, from cultivation to effective post-harvest measures, should be prioritized as future thrust areas. Moreover, the utilization of high-throughput genetic tools could be a significant boon for tropical ornamentals and researchers, facilitating exemplary improvements in post-harvest quality and addressing specific issues related to after-harvest handling of tropical cut flowers. This paradigm shift could position tropical cut flowers favorably in the global flower trade. Fruitful implementation, professional conduct, and openness to embrace innovation may transform this specialty group of cut flowers from an “unprofitable niche” into a remunerative and commercially viable component of the flower market.

The paved position of tropical flowers beyond boosting the iterated fact i.e global floral trade, can potently be useful in value-addition sector. Of late value-addition is one of the cardinal sectors of post-harvest industries, especially during the current ‘Go Green’ era. However, the multihued floral structures (like, inflorescences of tropical ornamental genotypes’, bi-color foliage of *H. metallica* etc.) and characteristics of a few botanical plant parts (like, fibrous pseudostem of *H. bihai* etc.) can endorse their eligibility to produce value-added products; as for example the production of eco-colorants and sources of natural cellulosic fibers. Furthermore, the botanical extracts of them may also be used for green synthesis of metallic nanoparticles (NPs) while for edible ornamental’s sector, they are almost unexplored. *Hitherto*, the rarest utilization of the members of specialty cut flowers in the said fields has been evidenced. In nutshell, considering the potential future prospects of these specialty TCFs, intensive scientific investigation may be a laudable approach.

## Author contributions

MM, PDOP, and MB conceived and conceptualized the article. The overall review of the entire paper has been made by MB, PDOP, and ARCN. Final compilation and editing were done by MM and MB. All authors contributed to the article and approved the submitted version.
